# Systematic metabolic analysis of potential target, therapeutic drug, diagnostic method and animal model applicability in three neurodegenerative diseases

**DOI:** 10.18632/aging.103253

**Published:** 2020-05-27

**Authors:** Wen-Xing Li, Gong-Hua Li, Xin Tong, Peng-Peng Yang, Jing-Fei Huang, Lin Xu, Shao-Xing Dai

**Affiliations:** 1 Key Laboratory of Animal Models and Human Disease Mechanisms, Kunming Institute of Zoology, Chinese Academy of Sciences, Kunming 650223, Yunnan, China; 2 Kunming College of Life Science, University of Chinese Academy of Sciences, Kunming 650204, Yunnan, China; 3 State Key Laboratory of Genetic Resources and Evolution, Kunming Institute of Zoology, Chinese Academy of Sciences, Kunming 650223, Yunnan, China; 4 Yunnan Key Laboratory of Primate Biomedical Research, Institute of Primate Translational Medicine, Kunming University of Science and Technology, Kunming 650500, Yunnan, China; 5 Centre for Excellence in Brain Science and Intelligent Technology, Chinese Academy of Sciences, Shanghai 200031, China

**Keywords:** neurodegenerative disease, metabolic damage, gene expression, drug, diagnostic model

## Abstract

Considerable evidence suggests that metabolic abnormalities are associated with neurodegenerative diseases. This study aimed to conduct a systematic metabolic analysis of Alzheimer’s disease (AD), Parkinson’s disease (PD) and Huntington’s disease (HD). Human and mouse model microarray datasets were downloaded from the Gene Expression Omnibus database. The metabolic genes and pathways were collected from the Recon 3D human metabolic model. Drug and target information was obtained from the DrugBank database. This study identified *ATP1A1*, *ATP6V1G2*, *GOT1*, *HPRT1*, *MAP2K1*, *PCMT1* and *PLK2* as key metabolic genes that were downregulated in AD, PD and HD. We screened 57 drugs that target these genes, such as digoxin, ouabain and diazoxide. This study constructed multigene diagnostic models for AD, PD and HD by using metabolic gene expression profiles in blood, all models showed high accuracy (AUC > 0.8) both in the experimental and validation sets. Furthermore, analysis of animal models showed that there was almost no consistency among the metabolic changes between mouse models and human diseases. This study systematically revealed the metabolic damage among AD, PD, and HD and uncovered the differences between animal models and human diseases. This information may be helpful for understanding the metabolic mechanisms and drug development for neurodegenerative diseases.

## INTRODUCTION

Neurodegenerative disease is characterized by progressive loss of structures and functions in brain and spinal cord neurons [1]. Common neurodegenerative diseases include Alzheimer's disease (AD), Parkinson's disease (PD), and Huntington's disease (HD). With the increase in global aging, the burden of these diseases is increasing rapidly worldwide [2]. AD is the most serious neurodegenerative disease, affecting approximately 0.6% of the global population [2]. The pathological features of AD include amyloid β and tau protein aggregation, mitochondrial dysfunction and synaptic injury [3, 4]. PD is the second most common neurodegenerative disease characterized by muscle stiffness, bradykinesia and uncontrollable tremors, and its severity causes gradual deterioration [5]. The main pathological anatomy of PD is the loss of large numbers of dopaminergic neurons in the substantia nigra [6]. HD is an autosomal dominant neurodegenerative disease, and the pathological feature is gradual degeneration of the striatal neurons, which affects muscle coordination and causes mental decline and psychopathological problems [7]. Mutations in the huntingtin (HTT) gene is the main cause of HD onset [8].

Multiple neurodegenerative diseases show severe metabolic abnormalities [9]. Damage from oxidative phosphorylation promotes AD, and it has been shown that oxidative damage occurs before Aβ deposition in APP transgenic mouse. The expression of energy metabolism-related genes is also affected in PD and HD [10]. Glutamate metabolism plays a crucial role in learning and memory, synaptic plasticity and neuronal development [11]. Abnormal glutamate metabolism causes neuronal dysfunction and degeneration in chronic neurodegenerative diseases [12]. Disorders of lipid metabolism are associated with AD and other neurodegenerative diseases. Impaired cholesterol metabolism will promote the processing of Aβ and lead to Aβ aggregation [13]. The citric acid cycle is a key link between sugar, lipid and amino acid metabolism and is an important process in energy metabolism. Studies have shown that damage to the citric acid cycle correlates with neurodegenerative disease pathology [14–16]. Lysosomal metabolic abnormalities can lead to decreased energy metabolism and a decreased clearance rate of cellular macromolecules, and studies have shown that dysfunction of lysosomal metabolism is correlated with AD, PD and HD [17].

The above evidence suggests that neurodegenerative diseases may share common metabolic damage. Therefore, the purpose of this study was to explore the common and differential metabolic damage in different brain regions among AD, PD and HD and to screen potential drugs that target the identified key metabolic genes. Furthermore, this study constructed multigene diagnostic models by using the expression profiles of metabolic genes in the blood. We also compared the metabolic differences between mouse models and human diseases.

## RESULTS

### Overall metabolic change in AD, PD and HD

Human brain transcriptome datasets of GSE5281 (AD) [18], GSE20295 (PD) [19] and GSE3790 (HD) [20] were collected for our reanalysis (Supplementary Table 1). There were no sex differences in any brain regions between cases and controls in these datasets. The age distribution in patients and controls showed no difference in most brain regions except for the PUT and SN in PD (Table 1). We mapped the metabolic genes from the Recon 3D human metabolism model [21] to the above datasets and performed differential expression gene analysis. The ratio of deregulated metabolic genes to total deregulated genes was higher than the ratio of mapped metabolic genes to all genes in most brain regions in the whole cohort, male and female groups, and in all brain regions in the elderly group (Supplementary Figure 1). This finding suggests that deregulated metabolic genes play important roles in these diseases. Through unsupervised clustering of all metabolic gene expression profiles, female patients and controls in the AD and PD datasets were mainly divided into two categories, and the other groups did not achieve the desired classification effect (Supplementary Figures 2–5). Interestingly, three brain regions in HD were divided into distinct classes, whereas there were no significant differences among brain regions in AD or PD in any groups. This indicates that the expression of metabolic genes in HD is brain-region specific.

**Table 1:** Sex and age information of neurodegenerative disease patients and controls.

**Neurodegenerative diseases**	**Sex (male/female)**			**Age (years)^1^**		
	**Case**	**Control**	**P^2^**	**Case**	**Control**	**P^3^**
*Alzheimer's Disease*						
Entorhinal Cortex	4/6	10/3	0.102	85.6 ± 6.3	80.3 ± 9.2	0.118
Hippocampus	6/4	10/3	0.650	77.8 ± 5.7	79.6 ± 9.4	0.574
Medial Temporal Gyrus	10/6	8/4	1.000	79.1 ± 6.4	80.1 ± 9.8	0.771
Posterior Cingulate	6/3	9/4	1.000	77.6 ± 6.5	79.8 ± 9.4	0.522
Superior Frontal Gyrus	13/10	7/4	1.000	79.2 ± 7.5	79.3 ± 10.2	0.977
Primary Visual Cortex	11/8	9/3	0.452	80.2 ± 6.7	77.9 ± 6.9	0.385
*Parkinson's Disease*						
Prefrontal Cortex	8/6	10/5	0.710	77.0 ± 6.3	71.2 ± 11.1	0.095
Putamen	9/6	15/5	0.467	76.7 ± 6.2	66.4 ± 13.8	0.006
Substantia Nigra	6/5	13/5	0.432	75.5 ± 5.8	66.8 ± 14.4	0.033
*Huntington's Disease*						
Caudate Nucleus	23/15	23/9	0.449	59.0 ± 14.9	58.4 ± 18.1	0.877
Frontal Cortex	22/15	19/9	0.606	56.6 ± 15.5	56.1 ± 17.5	0.914
Cerebellum	23/16	16/11	1.000	58.3 ± 15.6	59.1 ± 17.5	0.849

^1^ Data are presented as the mean ± standard deviation.

^2^ Fisher's exact test was used to compare population sex in the two groups.

^3^ Welch two sample t-test was used to compare population age in the two groups.

### Damaged metabolic pathways in AD, PD and HD

**Figure 1:**
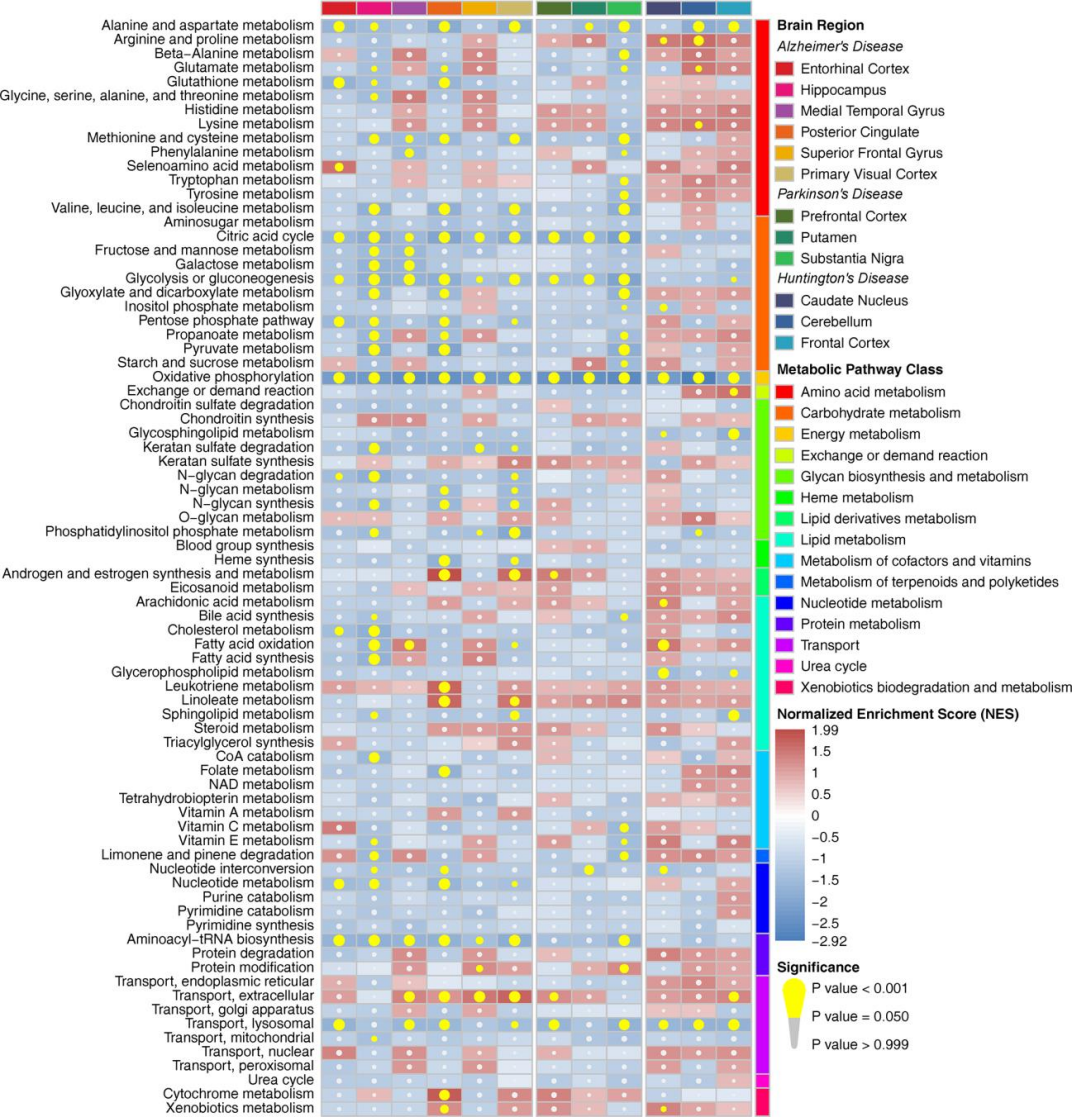
**Metabolic pathway enrichment results in three neurodegenerative diseases in the whole cohort.** The red box represents the metabolic pathway that is upregulated, and the blue box represents the metabolic pathway that is downregulated. The yellow circle indicates that the metabolic pathway is significantly enriched.

Metabolic pathway enrichment results showed that there were more impaired metabolic pathways in multiple brain regions in AD, whereas they were relatively less affected in PD and HD in the whole cohort (Figure 1). Alanine and aspartate metabolism, oxidative phosphorylation, extracellular transport and lysosomal transport were significantly enriched in multiple brain regions in all three diseases. Furthermore, most amino acid metabolism pathways and carbohydrate metabolism pathways were downregulated in at least one brain region in AD and PD (especially in the HIP and PC in AD and the SN in PD), whereas only a few of these metabolic pathways were affected in HD. We also observed a relatively consistent trend of metabolic pathway changes in the male, female and elderly groups (Supplementary Figures 6–8). Notably, male patients showed multiple downregulated metabolic pathways, whereas these pathways were nearly unaffected in the SN in female patients with PD. Pearson correlation analysis showed that there were strong positive correlations among pathways in amino acid metabolism, carbohydrate metabolism, nucleotide metabolism and protein metabolism, whereas pathways in energy metabolism, glycan biosynthesis and metabolism and lipid metabolism showed no or negative correlations with the above pathways in all brain regions (Supplementary Figure 9).

### Deregulated metabolic genes shared by multiple brain regions

**Figure 2:**
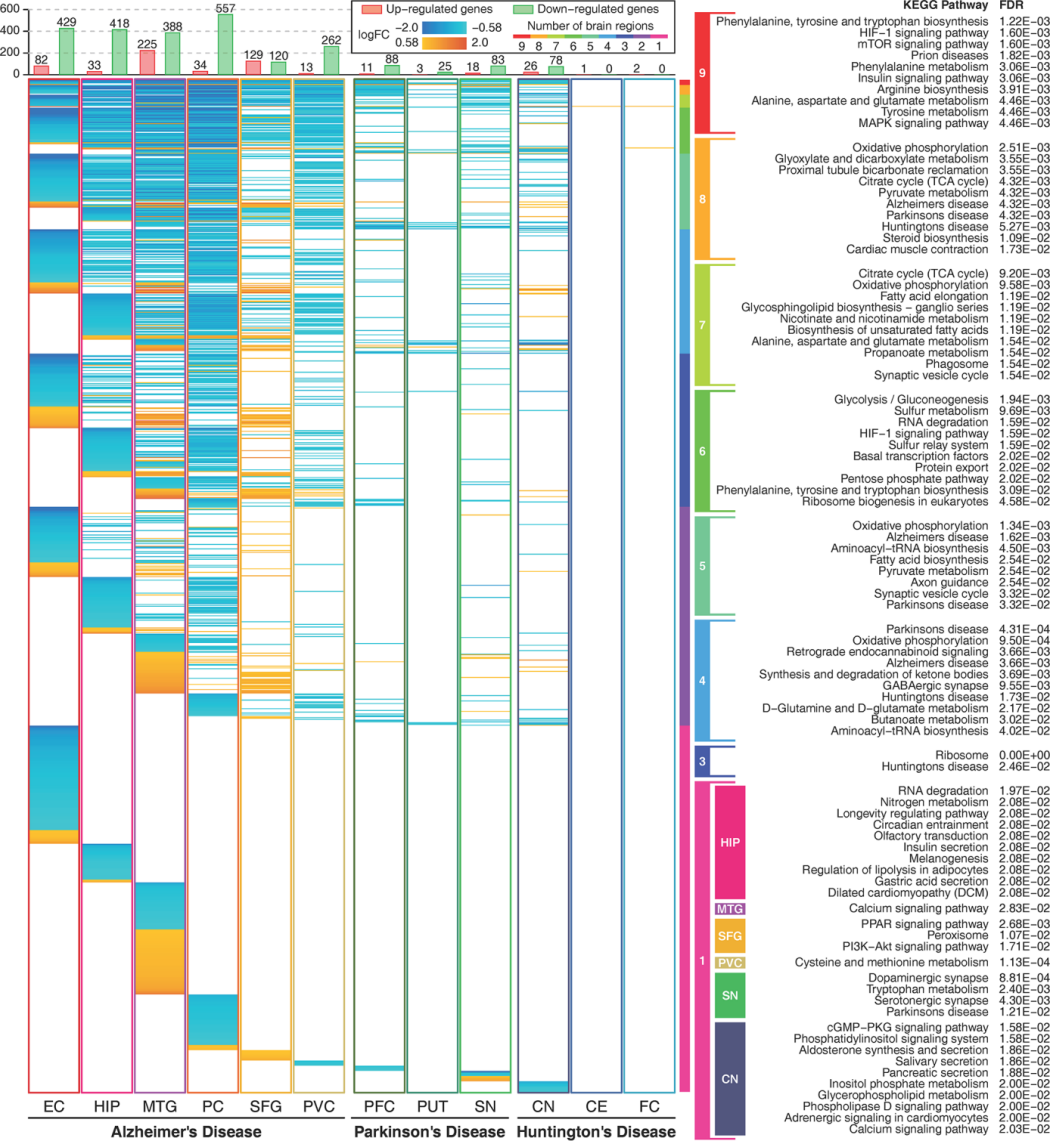
**Expression profiles of metabolic genes and their functions.** The figure shows metabolic genes with absolute logFC values higher than log2(1.5) in 12 brain regions. The figure shows 1164 unique metabolic genes. The orange color indicates that the gene is upregulated, and the cyan color indicates that the gene is downregulated. The rainbow color bar shows the commonly deregulated genes in multiple brain regions and their correlated metabolic pathways. Enriched metabolic pathways of brain region-specific deregulated genes are shown in colored boxes. Deregulated genes in two brain regions and deregulated genes only in the EC and the PC in AD, and the PFC in PD showed no significant enriched pathways.

We compared the commonly and heterogeneous deregulated metabolic genes for the three diseases. There were hundreds of deregulated metabolic genes in AD and relatively few deregulated metabolic genes in PD and HD. Most of these genes were downregulated in more than one brain region, and few upregulated genes were expressed in multiple brain regions (Figure 2). Deregulated metabolic genes shared by multiple brain regions were mostly enriched in amino acid metabolism, signaling transduction, carbohydrate metabolism, energy metabolism and several neurodegenerative disease-related pathways. This finding indicates that deregulated metabolic genes shared by multiple brain regions can accurately reflect the common characteristics of neurodegenerative diseases. Approximately one-third of these metabolic genes were deregulated only in one brain region, and these genes were enriched in relatively specific pathways. Furthermore, there were 92 heterogeneous deregulated genes (upregulated in one brain region and downregulated in another brain region), and most of these genes were found in AD (Supplementary Figure 10).

### Key metabolic genes in AD, PD and HD

**Figure 3:**
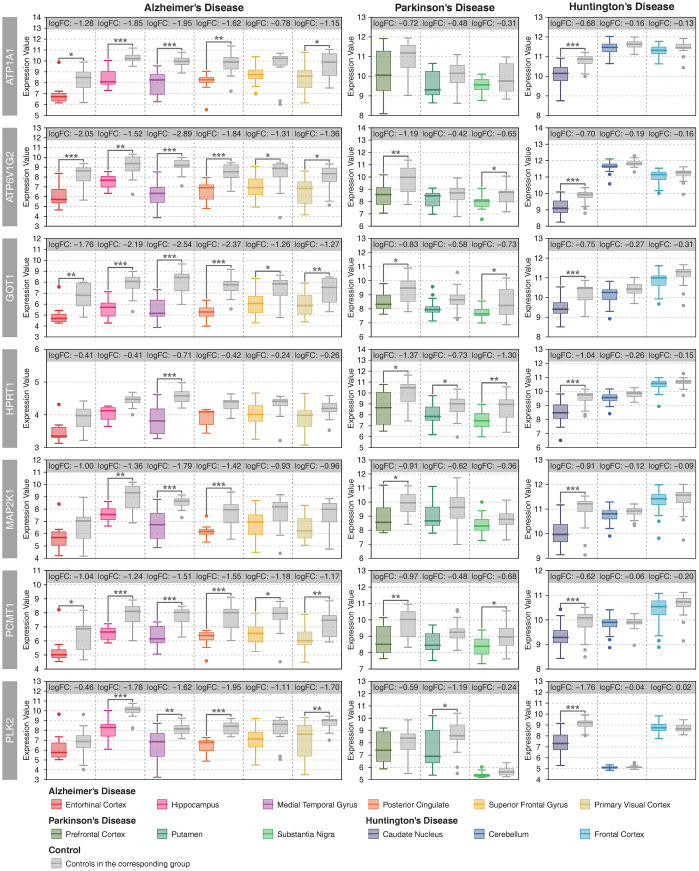
**Expression patterns of key metabolic genes.** Patient samples with different brain regions are represented by different colors, and the gray color represents the controls in the corresponding group. Student’s t-test was used to compare the expression differences between cases and controls. Statistical significance: * P < 0.05, ** P < 0.01, *** P < 0.001.

There were 40 deregulated metabolic genes shared by the three neurodegenerative diseases, most of which were consistently up- or downregulated in multiple brain regions (Supplementary Figure 11). The gene coexpression network showed that *ATP1A1*, *ATP6V1G2*, *GOT1*, *HPRT1*, *MAP2K1*, *PCMT1* and *PLK2* were significantly correlated with many other metabolic genes in AD, PD and HD (Supplementary Figure 12–14). Furthermore, the average degree of these metabolic genes was higher than 100 (Supplementary Figure 15). Therefore, we defined these genes as key metabolic genes. All of these genes were downregulated in multiple brain regions in AD and PD and in the CN in HD (Figure 3). A brain-specific network showed that these genes were mainly involved in nucleotide metabolic processes (Supplementary Figure 16). In recent years, the anti-aging gene *SIRT1* has been identified as an important metabolic gene that is critical to prevent metabolic diseases [22–25] and neurodegenerative diseases [26–28]. In this study, *SIRT1* was upregulated in the MTG and downregulated in the PC in AD whereas no difference in PD or HD (Supplementary Figure 17). Furthermore, there were strong positive correlations between *SIRT1* and key metabolic genes in the PVC in AD, and negative correlations between *SIRT1* and key metabolic genes in the MTG in AD, and the CN and the FC in HD (Supplementary Figure 17).

### Network of brain regions, metabolic pathways, key metabolic genes and drugs

**Figure 4:**
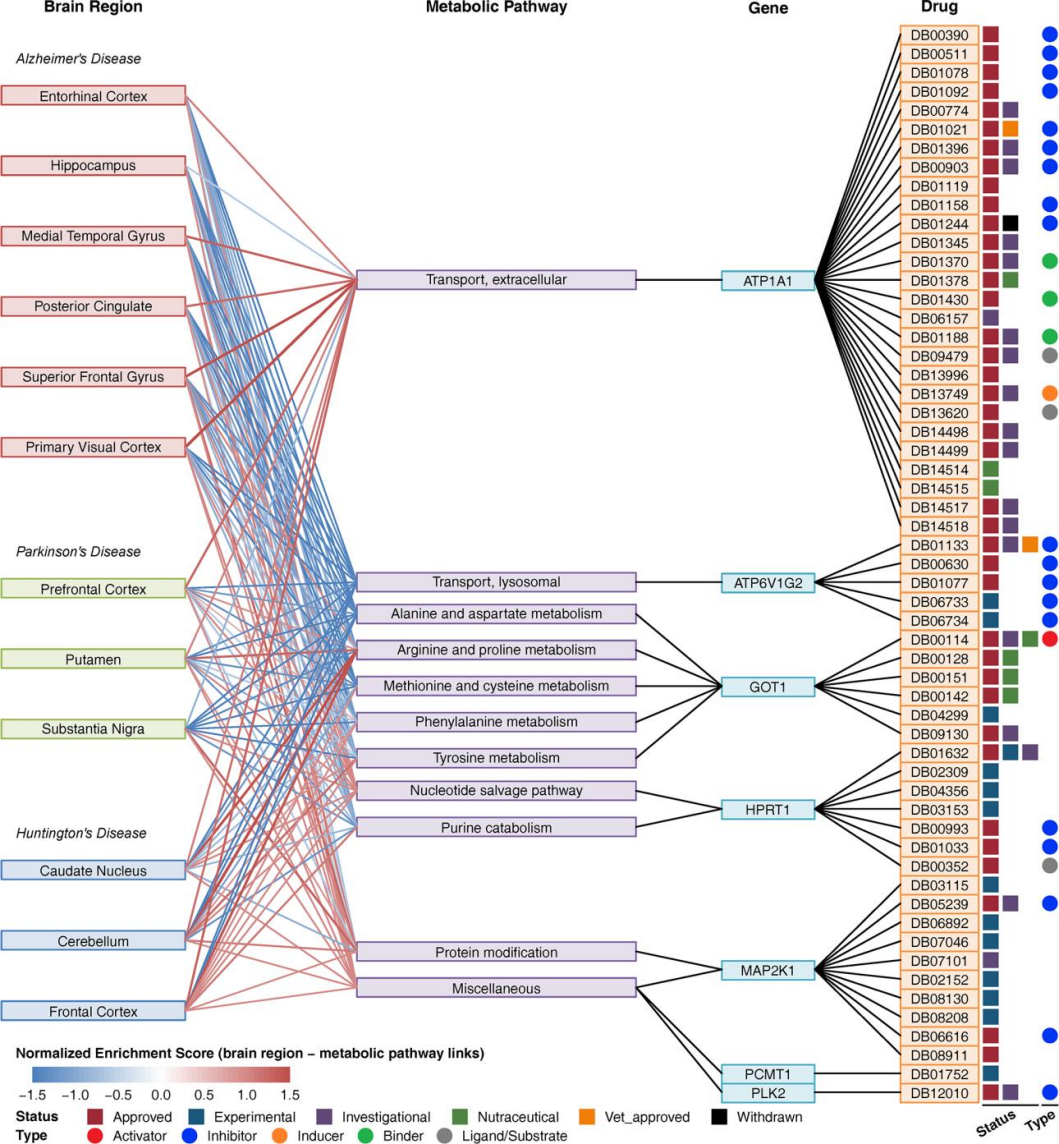
**Composite network of brain regions, metabolic pathways, key metabolic genes and drugs.** The link between brain region and metabolic pathway shows a normalized enrichment score of the pathway in the brain region. The red color indicates upregulation, and the blue color indicates downregulation. The link between metabolic pathways and genes indicates that the gene is involved in the pathway. The link between the gene and drug indicates that the drug can target the protein encoded by the gene. Drug status (approved, experimental, investigational nutraceutical, vet_approved, and withdrawn) is shown as colored squares. Drug type (activator, inhibitor, inducer, binder, ligand/substrate) is shown as colored circles.

Using the drug and target information from the DrugBank database [29], we constructed a composite network including brain regions, metabolic pathways, key metabolic genes and drugs (Figure 4). In this network, *ATP1A1* was involved in extracellular transport, and there were 27 drugs targeting *ATP1A1*. *ATP6V1G2* is involved in lysosomal transport, and there were 5 drugs targeting *ATP6V1G2*. *GOT1* is involved in multiple amino acid metabolism pathways, and there were 6 drugs targeting *GOT1*. Furthermore, there were 7 drugs targeting *HPRT1*, 10 drugs targeting *MAP2K1*, 1 drug targeting *PCMT1* and 1 drug targeting *PLK2*. No drug targeted multiple genes. Forty drugs were approved, and the others were experimental, investigational or nutraceutical drugs. Among these drugs, DB00114 is an activator of *GOT1*, and most of the other drugs are inhibitors of *ATP1A1*, *ATP6V1G2*, *HPRT1*, *MAP2K1* and *PLK2*. Many drugs targeting *ATP1A1* are used for cardiovascular disease treatment, drugs targeting *ATP6V1G2* are used for the treatment of osteoporosis and other bone diseases, drugs targeting *GOT1* are used for nutritional supplementation, and several drugs targeting *HPRT1* and *MAP2K1* are used for the treatment of immune-related disease and cancer (Supplementary Table 3).

### Multigene diagnostic models for AD, PD and HD

**Figure 5:**
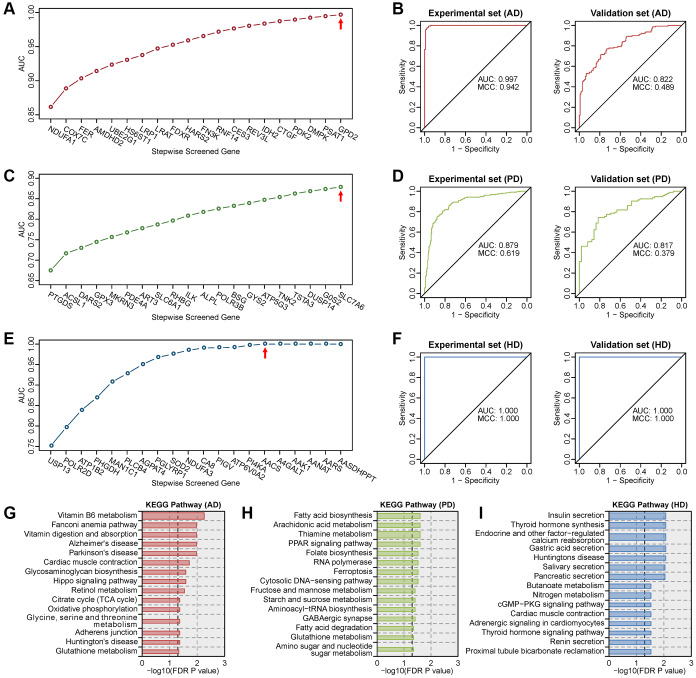
**Screening of the optimal multigene diagnostic model for three diseases.** (**A**) Stepwise screened multigene prediction models in AD. (**B**) Receiver operating characteristic (ROC) curves of the screened optimal diagnostic model in AD. (**C**) Stepwise screened multigene prediction models in PD. (**D**) ROC curves of the screened optimal diagnostic model in PD. (**E**) Stepwise screened multigene prediction models in HD. (**F**) ROC curves of the screened optimal diagnostic model in HD. For panels **A**, **C** and **E**, from left to right on the x-axis (stepwise screened genes), each additional gene corresponds to a model (for example, in panel **A**, *NDUFA1* represents model 1, which contains one gene, *NDUFA1*; *COX7C* represents model 2, which contains two genes including *NDUFA1* and *COX7C*). The red arrow shows the optimal model for each disease. Area under the curve (AUC) and Matthews correlation coefficient (MCC) were shown in the ROC curve. Details of the experimental set and validation set are provided in Supplementary Table 2. (**G**) Enriched KEGG pathway analysis of genes in the optimal diagnostic model for AD. (**H**) Enriched KEGG pathway analysis of genes in the optimal diagnostic model for PD. (**I**) Enriched KEGG pathway analysis of genes in the optimal diagnostic model for HD.

Expression profiles of metabolic genes in human blood transcriptome datasets (Supplementary Table 2) were used to construct multigene diagnostic models for AD, PD and HD. Multigene diagnosis models were built using the metabolic genes in the experimental set, and tested in the validation set. The optimal model for AD is the combination of 20 metabolic genes (Figure 5A), which had the highest AUC of 0.997 in the experimental set and a high AUC of 0.822 in the validation set (Figure 5B). The optimal model for PD is the combination of 20 metabolic genes (Figure 5C), which had the highest AUC of 0.879 in the experimental set and a high AUC of 0.817 in the validation set (Figure 5D). The optimal model for HD is the combination of 15 metabolic genes (Figure 5E), which reached the maximum AUC of 1.000 in both the experimental and validation sets (Figure 5F). The MCC values were high than 0.9 in AD experimental set, HD experimental and validation sets. KEGG enrichment results showed that genes in these diagnostic models were correlated with multiple neurodegenerative disease-related pathways (Figure 5G–5I). These results suggest that the expression profiles of metabolic genes in blood can be used for the highly accurate diagnosis of AD, PD and HD.

### Metabolic gene and pathway changes in mouse models

**Figure 6:**
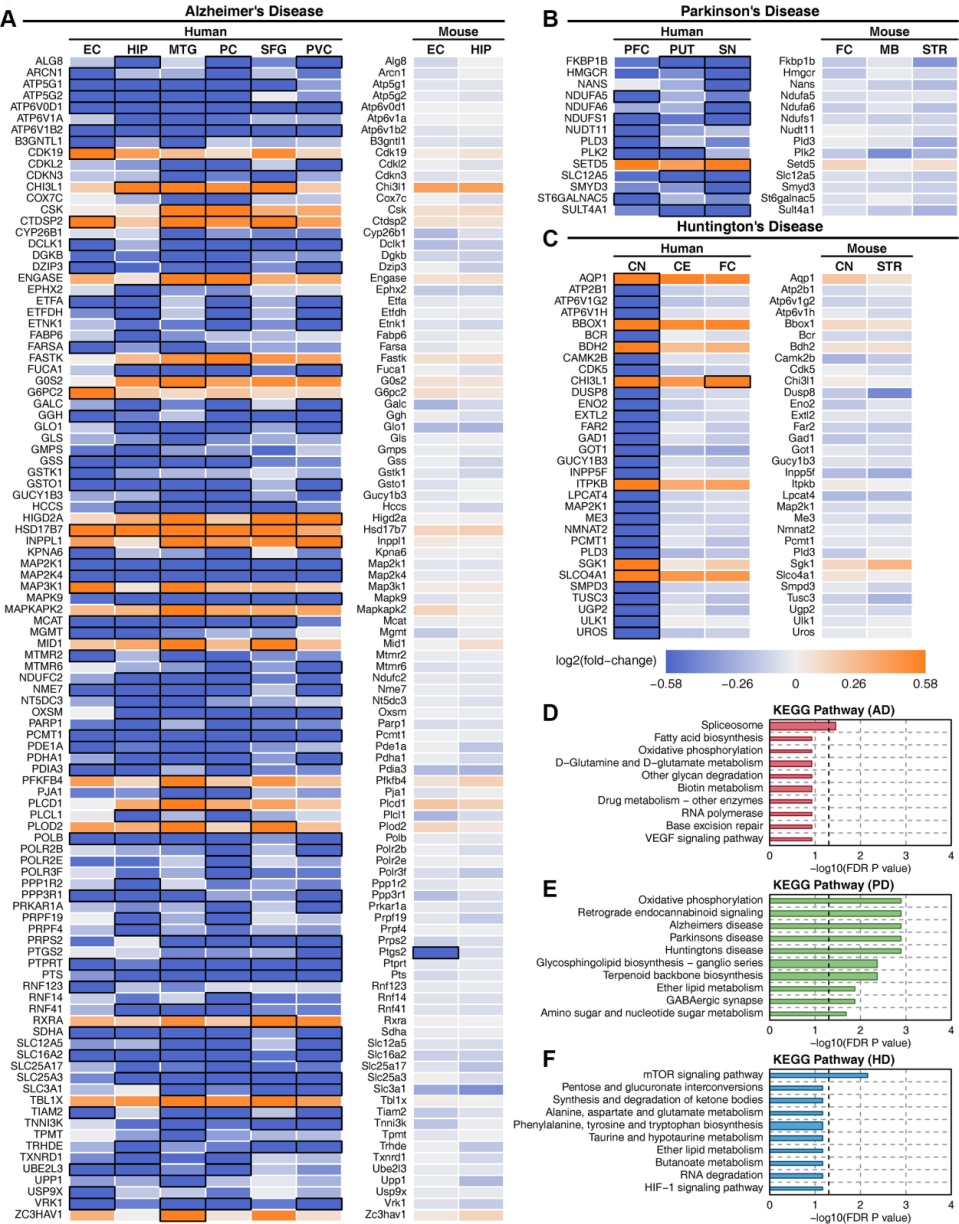
**Deregulated metabolic genes with consistent expression between human patients and mouse models.** (**A**) Heatmap of consistently expressed deregulated genes in AD human samples and the APP transgenic mouse model. (**B**) Heatmap of consistently expressed deregulated genes in PD human samples and the MPTP mouse model. (**C**) Heatmap of consistently expressed deregulated genes in HD human samples and the Hdh CAG knock-in mouse model. The orange color indicates that the gene is upregulated, the blue color indicates that the gene is downregulated, and black squares indicate statistical significance. (**D**) Enriched metabolic pathway of consistently expressed deregulated genes in AD. (**E**) Enriched metabolic pathway of consistently expressed deregulated genes in PD. (**F**) Enriched metabolic pathway of consistently expressed deregulated genes in HD.

There were 720, 1327 and 1024 metabolic genes that showed consistent expression trends in all brain regions in AD, PD and HD, respectively. We compared the metabolic changes between human diseases and mouse models (Supplementary Table 4). Deregulated metabolic genes were filtered in at least one brain region in each disease, and only 102, 14, and 32 metabolic genes showed the same expression trends in APP transgenic mouse, MPTP-treated mouse and Hdh CAG knock-in mouse models (Figure 6A–6C). However, almost all these genes showed no expression changes in mouse models. The functions of these metabolic genes were correlated with oxidative phosphorylation, GABAergic synapses and other neurodegenerative disease-related pathways in PD but not in AD or HD (Figure 6D–6F). Furthermore, metabolic pathway enrichment results showed that amino acid metabolism, carbohydrate metabolism, energy metabolism and other metabolic pathways that were severely impaired in human patients were only slightly affected in mouse models (Supplementary Figure 18). The expression of key metabolic genes in mouse models also showed no difference (Supplementary Figure 19). These results suggest that mouse models cannot accurately reflect human metabolic characteristics in neurodegenerative diseases.

## DISCUSSION

Although there were different degrees of metabolic damage in AD, PD and HD, most metabolic genes and pathways showed consistent downregulated trends in these three diseases, and fewer genes were expressed inconsistently in different brain regions. This study identified *ATP1A1*, *ATP6V1G2*, *GOT1*, *HPRT1*, *MAP2K1*, *PCMT1* and *PLK2* as key metabolic genes in AD, PD and HD. *ATP1A1* encodes subunit alpha 1 of Na^+^/K^+^-ATPase, which is crucial for establishing and maintaining the electrochemical gradients of Na and K ions across the plasma membrane. Decreased levels of Na^+^/K^+^-ATPase cause energy deficiency in multiple neurodegenerative diseases [30]. *ATP6V1G2* encodes subunit G2 of vacuolar ATPase (V-ATPase), which transports protons from the cytoplasm into the lysosome and maintains lysosomal acidification. V-ATPase deficiency can lead to central nervous system (CNS) diseases such as AD and PD [31, 32]. *GOT1* encodes glutamic oxaloacetic transaminase in the cytoplasm, and downregulated *GOT1* was found both in the elderly population and AD patients [33]. *HPRT1* encodes hypoxanthine phosphoribosyltransferase 1, and mutated *HPRT1* affects amyloid precursor protein (APP) gene expression in AD and amyotrophic lateral sclerosis (ALS) [34]. *MAP2K1* regulates a wide variety of extra- and intracellular signals. The compromised MAPK signaling pathways contribute to the pathology of diverse human diseases, including cancer and neurodegenerative disorders such as AD, PD and ALS [35]. *PCMT1* plays a role in protein repair; downregulated *PCMT1* expression makes it difficult to repair proteins involved in apoptosis and could contribute to the neuronal cell death observed in PD [36]. *PLK2* is a homeostatic repressor of neuronal overexcitation, which promotes APP β-processing in AD [37] and catalyzes α-synuclein in PD [38].

This study identified 57 drugs that target the above key metabolic genes. Digoxin (DB00390) is an endogenous inhibitor of membrane Na^+^/K^+^-ATPase, which is used to treat chronic atrial fibrillation and mild to moderate heart failure. Molecular docking showed that digoxin may regulate metabolic functions in AD by combining with G protein-coupled receptors [39]. A previous clinical trial showed that digoxin has a good effect on the treatment of PD [40]. Furthermore, serum digoxin can regulate neutral amino acid transport and mitochondrial functions in HD patients [41]. Ouabain (DB01092) is able to inhibit Na^+^/K^+^-ATPase activity in multiple brain regions [42]. Animal experiments have shown that ouabain induces downstream autophagy-lysosomal gene expression and cellular restorative properties and reduces the accumulation of abnormal toxic tau protein [43]. Diazoxide (DB01119) is mainly used to treat hyperinsulinemic hypoglycemia, and animal experiments have shown that diazoxide can also be used in the treatment of PD [44]. There was no report on the treatment of neurodegenerative diseases for most of the drugs screened in this study. Therefore, further investigation of these drugs for the treatment of AD, PD and HD is worthwhile.

There are still tremendous difficulties in building reliable and reproducible diagnostic models for neurodegenerative diseases. Increasing research suggests that the blood transcriptome signature may enable accurate diagnosis of these diseases. A recent study showed that the combination of a multitissue RNA signature can accurately diagnose AD and other aging-related diseases [45]. Many studies have reported that blood-based biomarkers could be potential predictors for PD, such as α-synuclein, DJ-1, and uric acid [46], and blood-based gene signatures also showed high accuracy in PD diagnosis [47]. Furthermore, previous studies showed that the gene signature in peripheral blood can be used for accurate diagnosis of HD [48, 49]. This study constructed multigene diagnostic models for AD, PD and HD by using metabolic gene expression profiles in blood. All models showed high accuracy both in the experimental set and validation set. Therefore, the diagnosis of neurodegenerative diseases using metabolic gene signatures in blood may be an effective method.

The mouse model is the most widely used animal model in neurodegenerative disease studies and can partially reflect the behavioral, pathological and genetic characteristics of human diseases [50]. Rodents do not develop AD, and the existing AD transgenic mouse models can only reflect limited human disease characteristics [51]. There are large differences in gene expression signatures in neuroimmune and neurodegenerative pathways between human and APP transgenic mouse models [52]. The gene expression profiles of microglial activation states in AD patients are not apparent in mouse models [53]. Furthermore, a meta-analysis including 33 microarray studies of PD shows that consistent features in human datasets are not shown in mouse models [54]. Fortunately, the HD mouse model is relatively successful and can model early-onset states in humans [55]. This study revealed almost no consistency of metabolic changes between human neurodegenerative disease patients and mouse models. Therefore, we speculate that mouse models may not be suitable for studying the metabolic mechanisms of neurodegenerative diseases.

In conclusion, there was severe metabolic damage in AD, PD and HD. Most metabolic damage, such as amino acid metabolism, carbohydrate metabolism, energy metabolism and multiple transport metabolism, is common to all three diseases. We identified 7 key metabolic genes that were downregulated in all three diseases and screened 57 drugs that target these genes. Some drugs have been reported to be effective in the treatment of neurodegenerative diseases. Furthermore, metabolic gene expression profiles in blood can be used for the diagnosis of AD, PD and HD. This study also found considerable metabolic differences between mouse models and human diseases.

## MATERIALS AND METHODS

### Neurodegenerative disease data collection

Microarray datasets of AD, PD and HD were downloaded from the Gene Expression Omnibus (GEO) database (http://www.ncbi.nlm.nih.gov/geo/). We conducted rigorous screening of these datasets with the following inclusion criteria: (1) the human microarray datasets were genome-wide; (2) samples in each study should include cases and controls; (3) each dataset should contain multiple brain regions; and (4) raw data or expression matrixes were available. Because these neurodegenerative diseases may be affected by age and sex factors, we tried to screen the datasets without age or sex bias between patients and controls. According to the above criteria, we finally chose GSE5281 (AD) [18], GSE20295 (PD) [19] and GSE3790 (HD) [20] for our reanalysis (Supplementary Table 1). For details on data preprocessing, see our previous reports [56, 57]. The brain regions in the AD dataset include the entorhinal cortex (EC), hippocampus (HIP), medial temporal gyrus (MTG), posterior cingulate (PC), superior frontal gyrus (SFG), and primary visual cortex (PVC). The brain regions in the PD dataset include the prefrontal cortex (PFC), putamen (PUT), and substantia nigra (SN). The brain regions in the HD dataset include the caudate nucleus (CN), cerebellum (CE) and frontal cortex (FC). Considering the potential effects of sex and age, we analyzed the metabolic changes in the whole cohort, male, female, and elderly (age ≥ 60 years) groups.

### Metabolic gene collection

Human metabolic genes were extracted from the Recon 3D human metabolism model [21]. This model contains 3,288 metabolic genes that belong to 105 metabolic pathways. Due to the different analysis platforms of the datasets we collected, we screened the metabolic genes shared by all datasets for analysis. In total, we mapped 2455 unique metabolic genes in our datasets.

### Differential expression gene analysis

Bioinformatics analysis of the microarray data was carried out by R statistical software v3.6.1 and Bioconductor Library. Differential gene expression analysis was performed using the empirical Bayesian algorithm in the limma package in R [58]. Up- and downregulated genes were defined as a log2 transformed fold-change (logFC) ≥ log2(1.5) or ≤ log2(1/1.5) for patients compared with controls. A false discovery rate (FDR)-corrected P value ≤ 0.05 was considered significant. The pheatmap package in R was used to show the gene expression profiles, and the clustering method was chosen as "ward.D2".

### Metabolic pathway enrichment analysis

We used javaGSEA desktop application v3.0 to perform gene set enrichment analysis (GSEA) of affected metabolic pathways for a total of 12 brain regions in AD, PD and HD. The extracted metabolic genes and pathways from Recon3D were used to construct gene sets for enrichment analysis. Gene sets with fewer than 10 genes or more than 500 genes were excluded. The t-statistic mean of the genes was computed for each metabolic pathway using a permutation test with 1000 replications. Up- and downregulated metabolic pathways were defined as a normalized enrichment score (NES) > 0 or < 0 for patients compared with controls. An FDR-corrected P value ≤ 0.05 was considered significant.

### Coexpression network analysis and key metabolic gene screen

Pearson’s correlation coefficient was calculated for each gene-gene pair of all metabolic genes in AD, PD and HD. Gene-gene pairs with an absolute value of correlation coefficient higher than 0.75 and an FDR-corrected P value ≤ 0.05 were considered significantly correlated. Significant gene-gene pairs were used to construct the gene coexpression networks. The selection criteria for key metabolic genes are as follows: (1) The absolute of logFC of metabolic genes is higher than log2(1.5) in at least one brain region in each disease. (2) The average number of nodes of metabolic genes in gene coexpression networks is higher than 100.

### Brain-specific gene network analysis

Brain-specific gene network analysis was performed using the HumanBase web server (https://hb.flatironinstitute.org/) [59]. The screened key metabolic genes were used as input genes to perform the gene network analysis. The tissue option in parameter settings was chosen as the brain, and the data types option included coexpression, interaction, TF binding and GSEA perturbations. The minimum interaction confidence and the maximum number of genes were determined using default settings. The server generated a gene network of the queried genes and other genes that interacted with these genes, and GO biological process enrichment analysis of the genes in the network was performed.

### Drug discovery and composite network construction

Drugs that interact with the screened key metabolic genes were searched from the DrugBank database (https://www.drugbank.ca/) [29]. Information was obtained on the ID, name, status, types, and indication/associated conditions of the screened drugs. Then, we constructed a composite network of 12 brain regions in AD, PD and HD, deregulated metabolic pathways, key metabolic genes and drugs.

### Blood transcriptome analysis and multigene diagnosis model

To investigate the effects of metabolic gene expression profiles in disease diagnosis, we downloaded blood transcriptome datasets of AD, PD and HD from the GEO database. Each disease contains an experimental dataset and a validation dataset (Supplementary Table 2). Multigene diagnosis models were built using the metabolic genes in the experimental set, and the validation set was used to test the predictive accuracy of the model. A univariate logistic regression model was used to calculate the odds ratios of the metabolic genes in each disease. The receiver operating characteristic (ROC) curve and the area under the curve (AUC) of the single metabolic genes were calculated using the pROC package in R. The model with the largest AUC was defined as the optimal model. A stepwise modeling strategy was used to screen the optimal multigene combination models for each disease. The maximum number of metabolic genes in the model is set to 20. First, the gene with the largest AUC was selected. Then, we used a multivariate logistic regression model to generate the combined effect of the selected gene and each of the remaining genes. Next, we selected the best two-gene model with the highest AUC and repeated the previous steps. Finally, we selected the optimal model with the highest AUC in each multigene combination model. Matthews correlation coefficient (MCC) [60] was calculated for each optimal model.

### Mouse model analysis

Since mouse models are widely used in the study of neurodegenerative diseases, we analyzed the changes in metabolic genes and pathways in three mouse models (APP transgenic mouse for AD, MPTP-treated mouse for PD and Hdh CAG knock-in mouse for HD) and compared them with human disease. Datasets of mouse models were downloaded from the GEO database. Each mouse model contains transcriptome data for multiple brain regions (Supplementary Table 4). We screened metabolic genes with consistent expression trends in all brain regions in each disease and compared the expression changes of these genes in mouse models.

## Supplementary Material

Supplementary Figures

Supplementary Tables 1, 2, 4

Supplementary Table 3
