# Supplementary material for: Systematic metabolic analysis of potential target, therapeutic drug, diagnostic method and animal model applicability in three neurodegenerative diseases
Source: Aging (Albany NY). 2020 May 27;12(10):9882–914. doi: 10.18632/aging.103253 (PMC7288927; doi:10.18632/aging.103253)
Supplement: Supplementary Figures [file aging-12-103253-s003..pdf]

SUPPLEMENTARY FIGURES

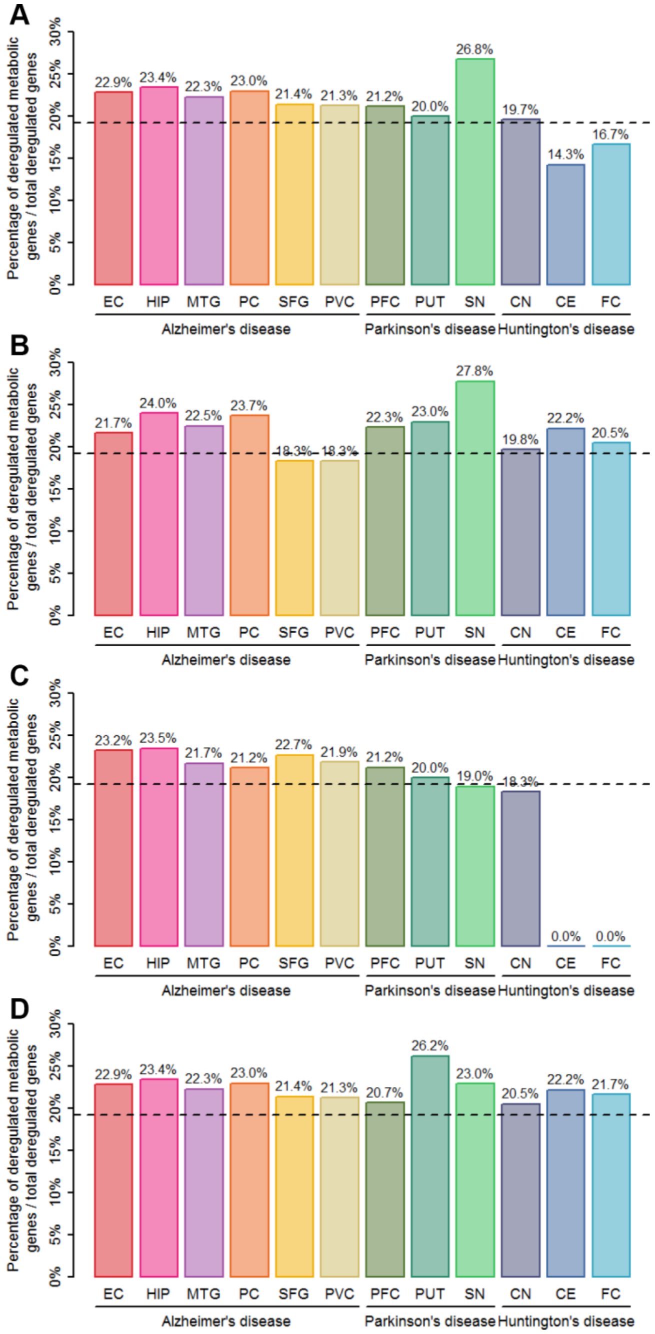

Supplementary Figure 1. The percentage of deregulated metabolized genes to total deregulated genes in whole cohort. (**A**), male group (**B**), female group (**C**) and elderly group (**D**). The elderly group was defined as the age ≥ 60 years. The dashed line indicates the percentage of metabolic genes in total genes. Each color represents a brain region.

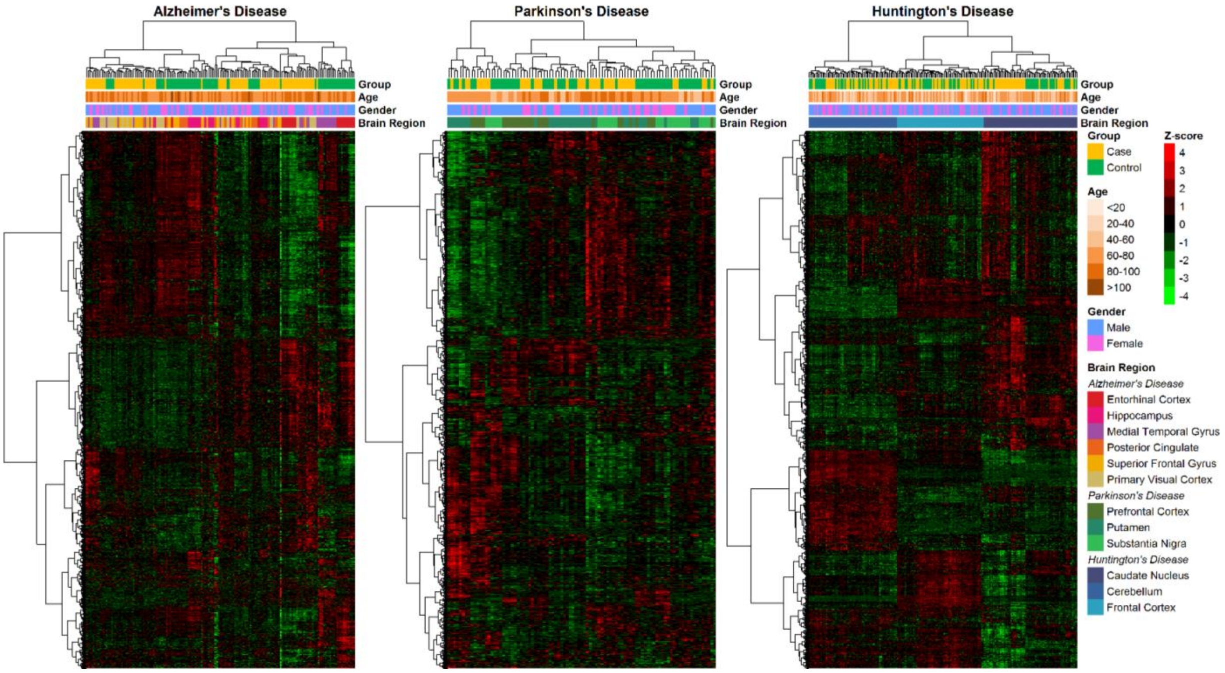

Supplementary Figure 2. Metabolic genes clustering of three neurodegenerative diseases in whole cohort. A total of 2455 metabolic genes were included in the heatmap. All gene expression values were z-score converted. The clustering method was chosen as "ward.D2".

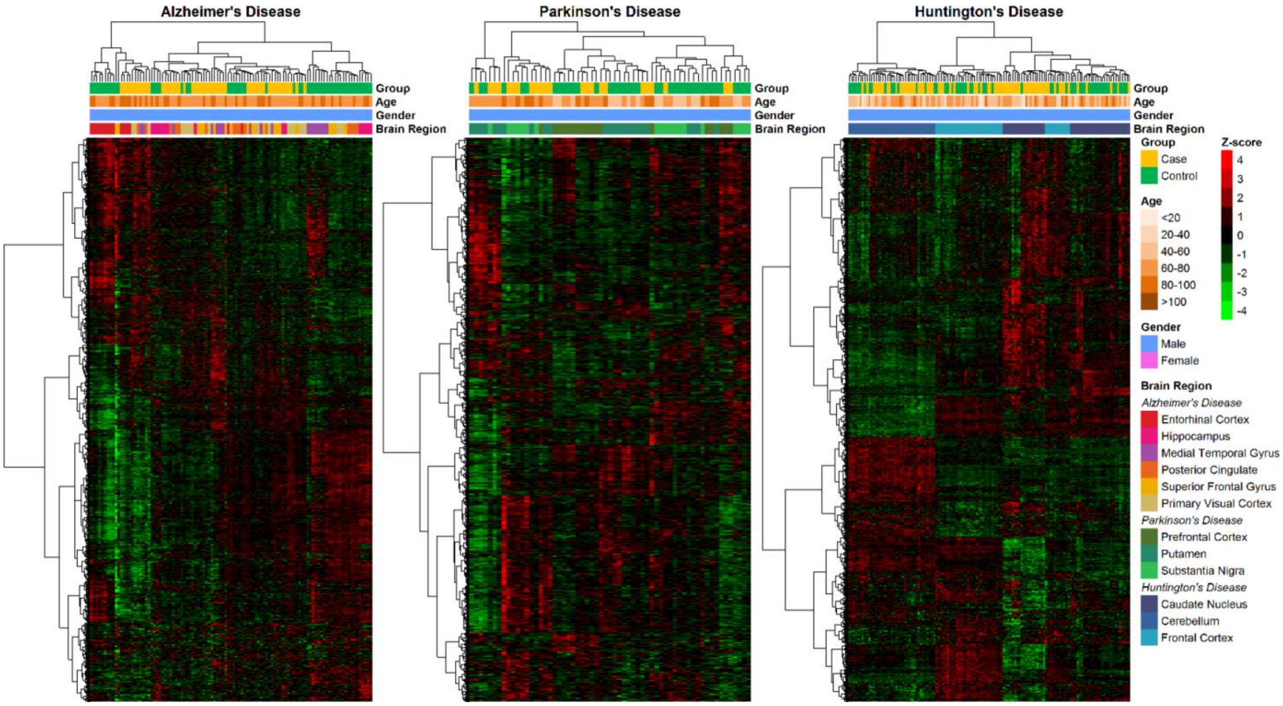

Supplementary Figure 3. Metabolic genes clustering of three neurodegenerative diseases in male group. A total of 2455 metabolic genes were included in the heatmap. All gene expression values were z-score converted. The clustering method was chosen as "ward.D2".

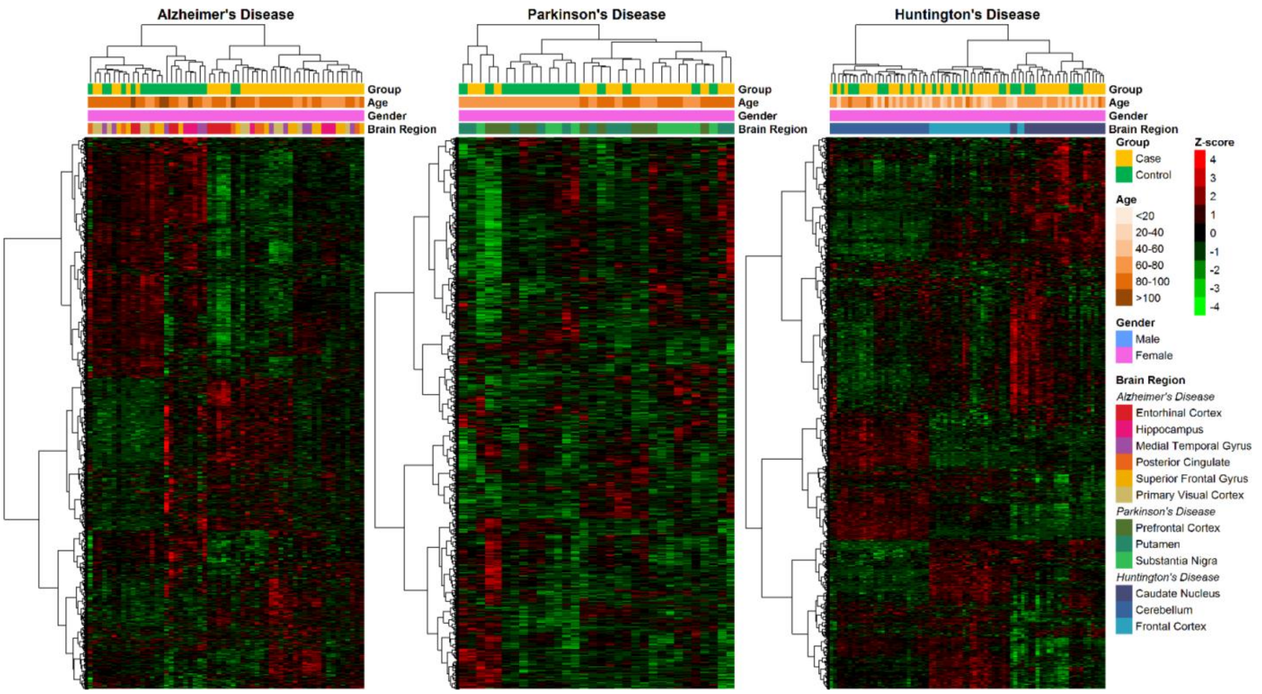

Supplementary Figure 4. Metabolic genes clustering of three neurodegenerative diseases in female group. A total of 2455 metabolic genes were included in the heatmap. All gene expression values were z-score converted. The clustering method was chosen as "ward.D2".

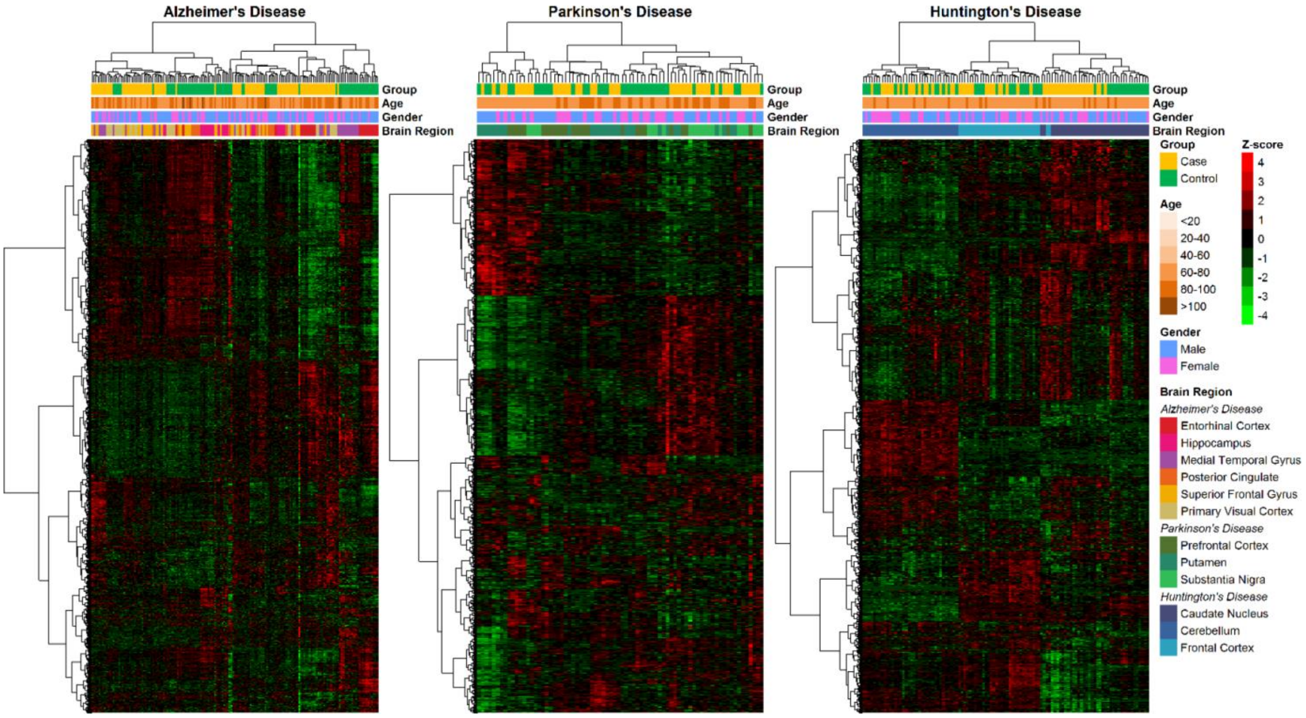

Supplementary Figure 5. Metabolic genes clustering of three neurodegenerative diseases in elderly group. The elderly group was defined as the age ≥ 60 years. A total of 2455 metabolic genes were included in the heatmap. All gene expression values were z-score converted. The clustering method was chosen as "ward.D2".

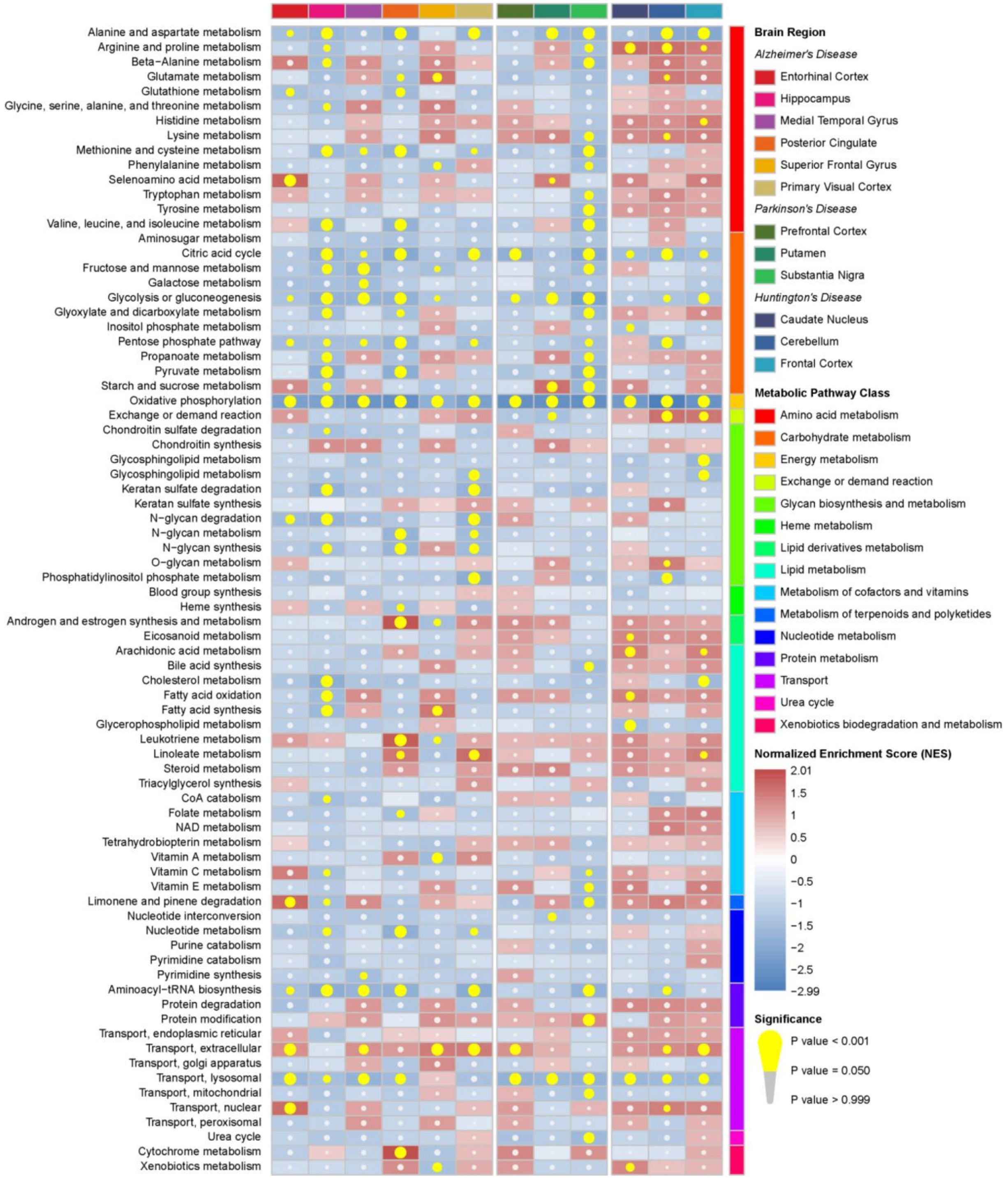

Supplementary Figure 6. Metabolic pathway enrichment results in three neurodegenerative diseases in male group. The red box represents the metabolic pathway is up-regulated and the blue box represents the metabolic pathway is down-regulated. The yellow circle indicates the metabolic pathway is significantly enriched.

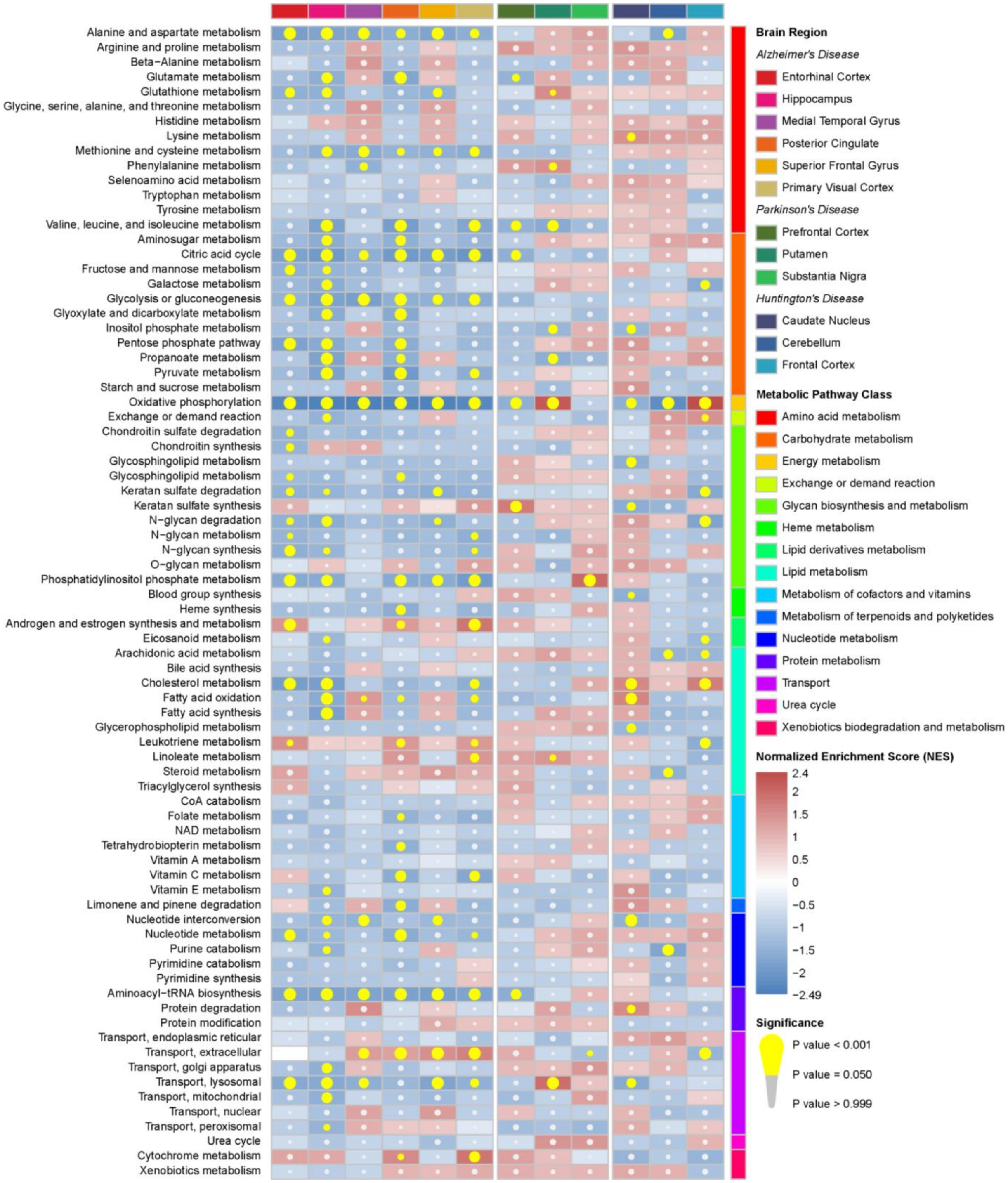

Supplementary Figure 7. Metabolic pathway enrichment results in three neurodegenerative diseases in female group. The red box represents the metabolic pathway is up-regulated and the blue box represents the metabolic pathway is down-regulated. The yellow circle indicates the metabolic pathway is significantly enriched.

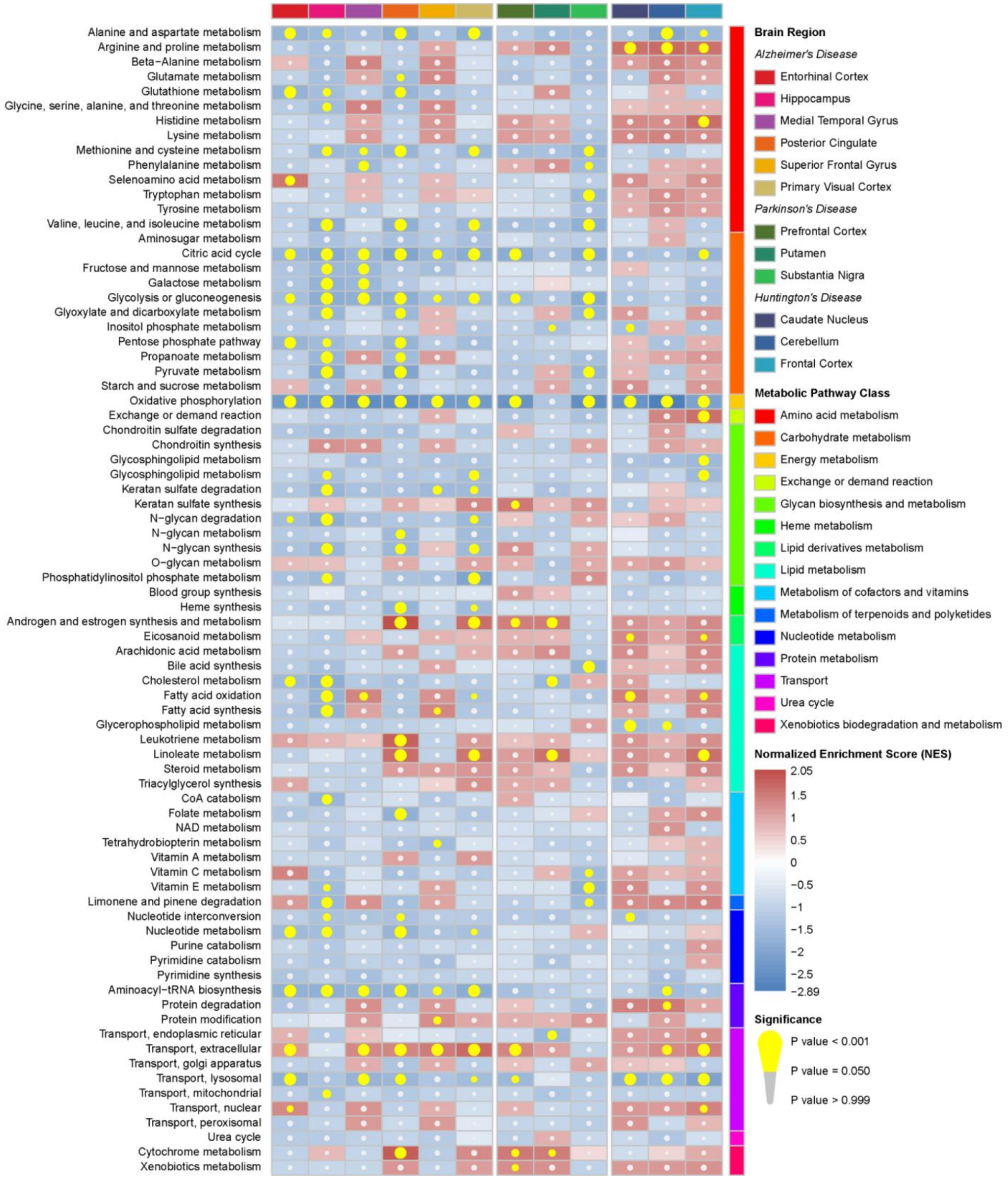

Supplementary Figure 8. Metabolic pathway enrichment results in three neurodegenerative diseases in elderly group. The elderly group was defined as the age ≥ 60 years. The red box represents the metabolic pathway is up-regulated and the blue box represents the metabolic pathway is down-regulated. The yellow circle indicates the metabolic pathway is significantly enriched.

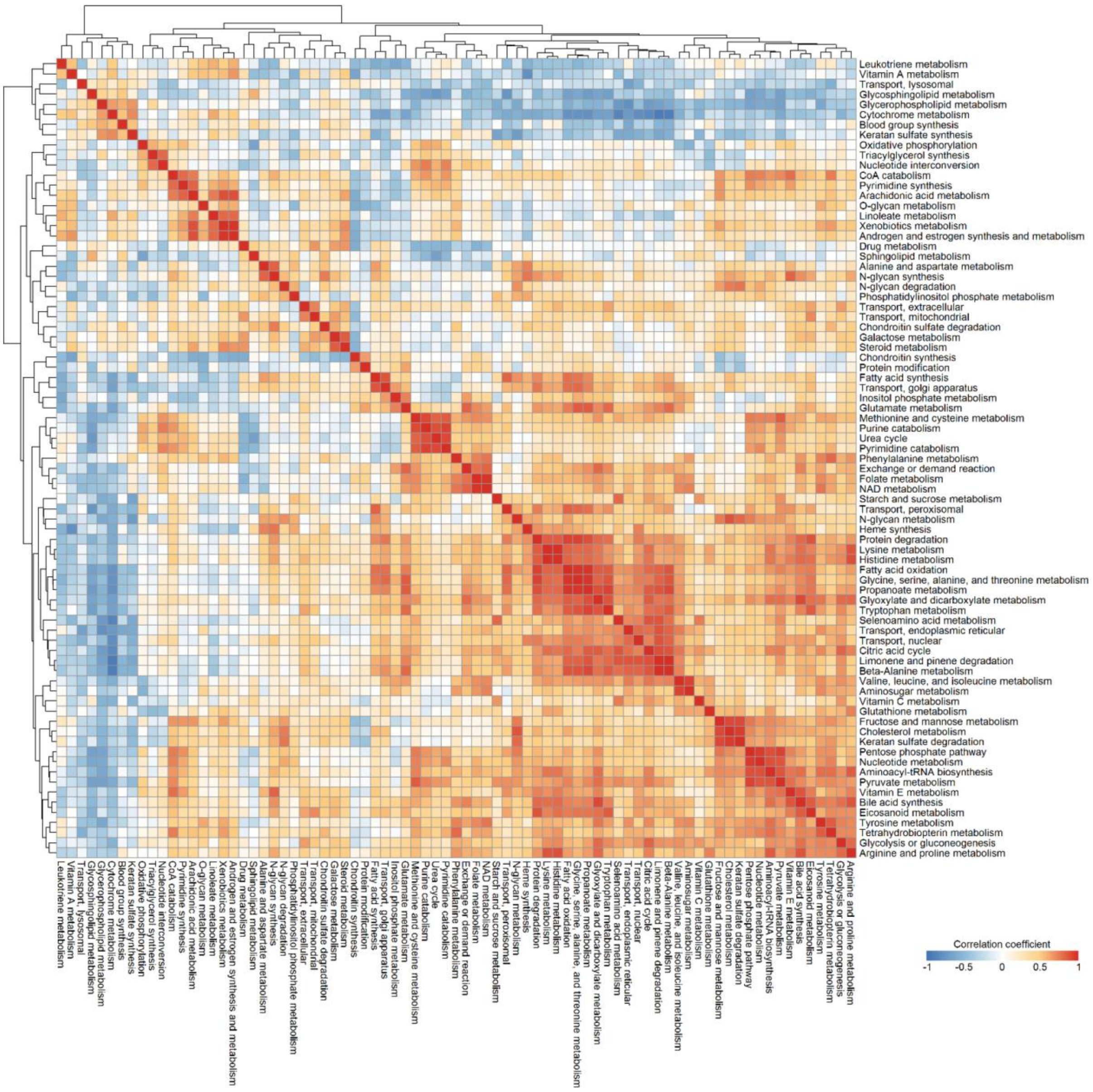

Supplementary Figure 9. Correlation of metabolic pathway changes in three neurodegenerative diseases. Pearson's correlation coefficient was calculated using normalized enrichment scores of metabolic pathways in all brain regions. The positive correlation indicates consistent changes in metabolic pathways and the negative correlation indicates opposite changes in metabolic pathways.

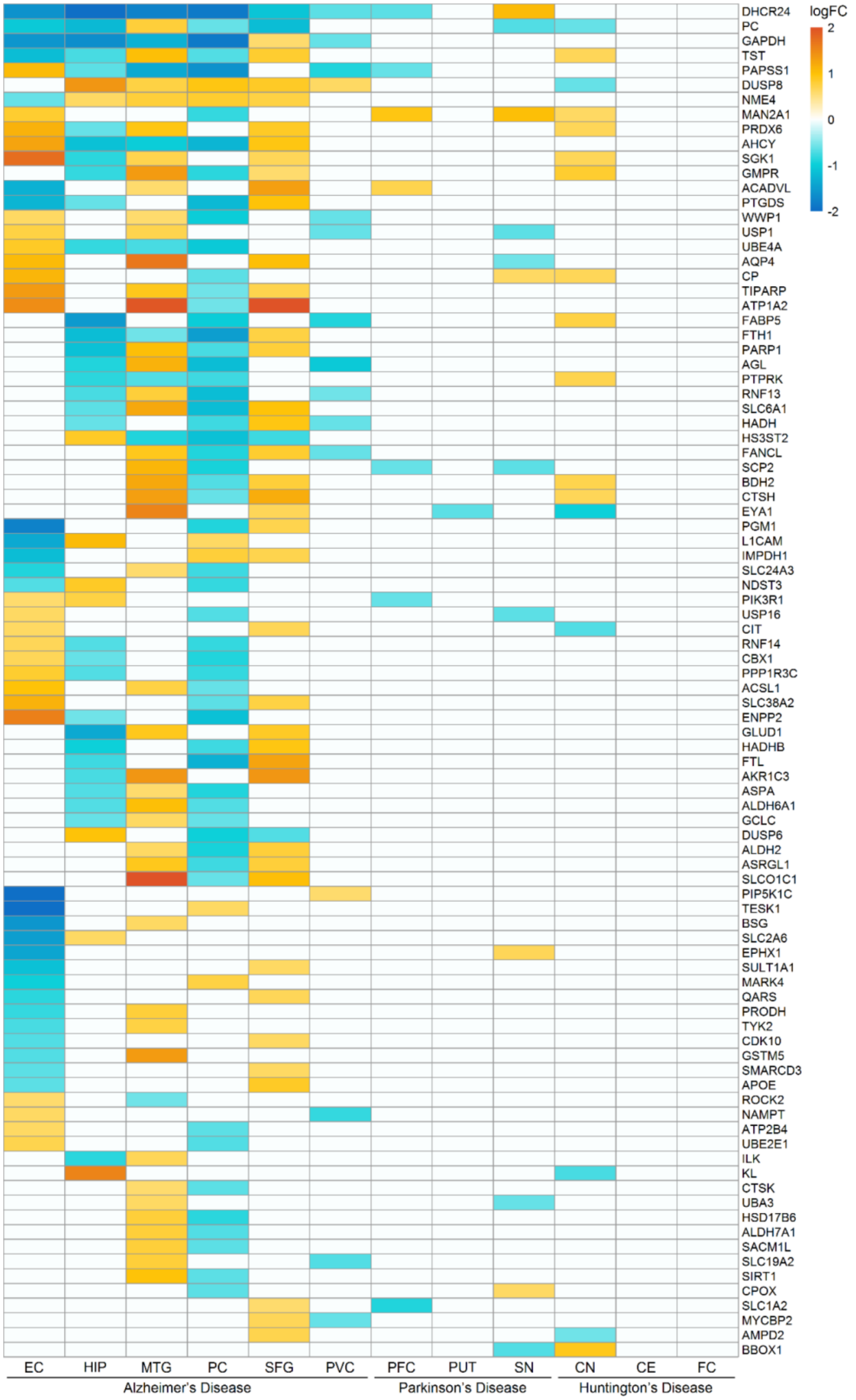

Supplementary Figure 10. Expression of heterogeneous deregulated genes in three neurodegenerative diseases. The figure showed 92 metabolic genes. Genes that are upregulated in one brain region and downregulated in another brain region are defined as heterogeneous deregulated genes. The heatmap showed genes with the absolute value of logFC higher than log2(1.5). Genes with logFC below threshold are not shown the expression profiles.

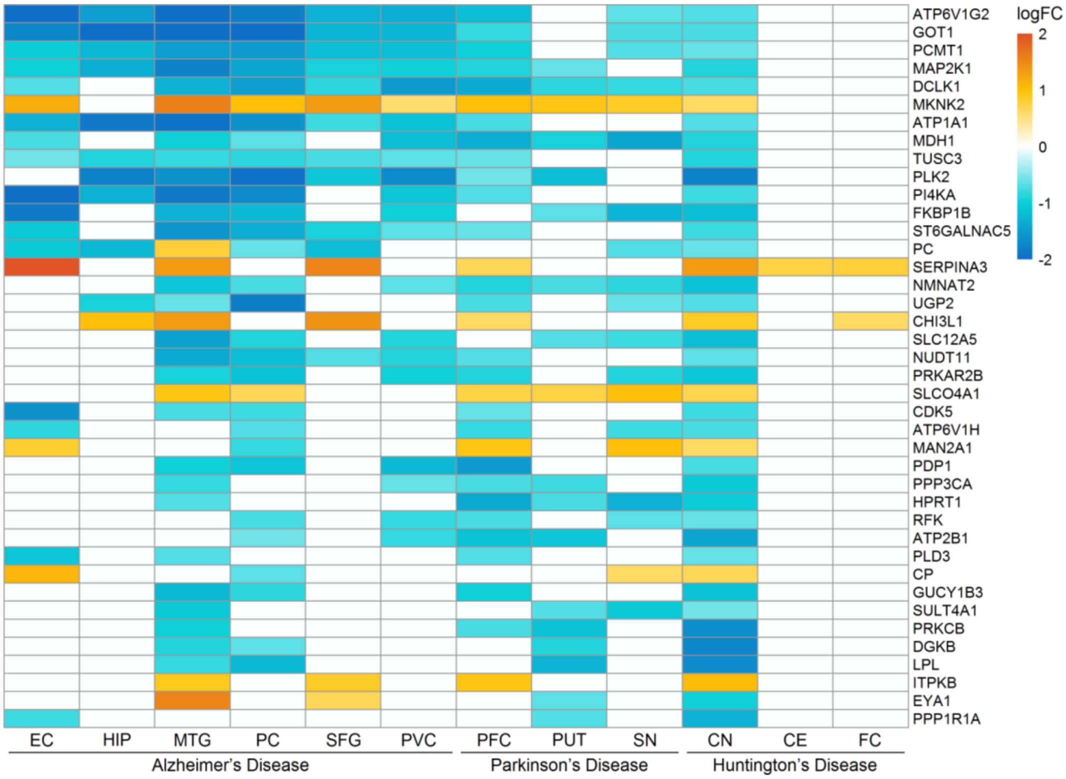

Supplementary Figure 11. Expression of deregulated genes shared by three neurodegenerative diseases. The heatmap showed genes with the absolute value of logFC higher than log2(1.5) in at least one brain region in each disease. Genes with logFC below threshold are not shown the expression profiles.

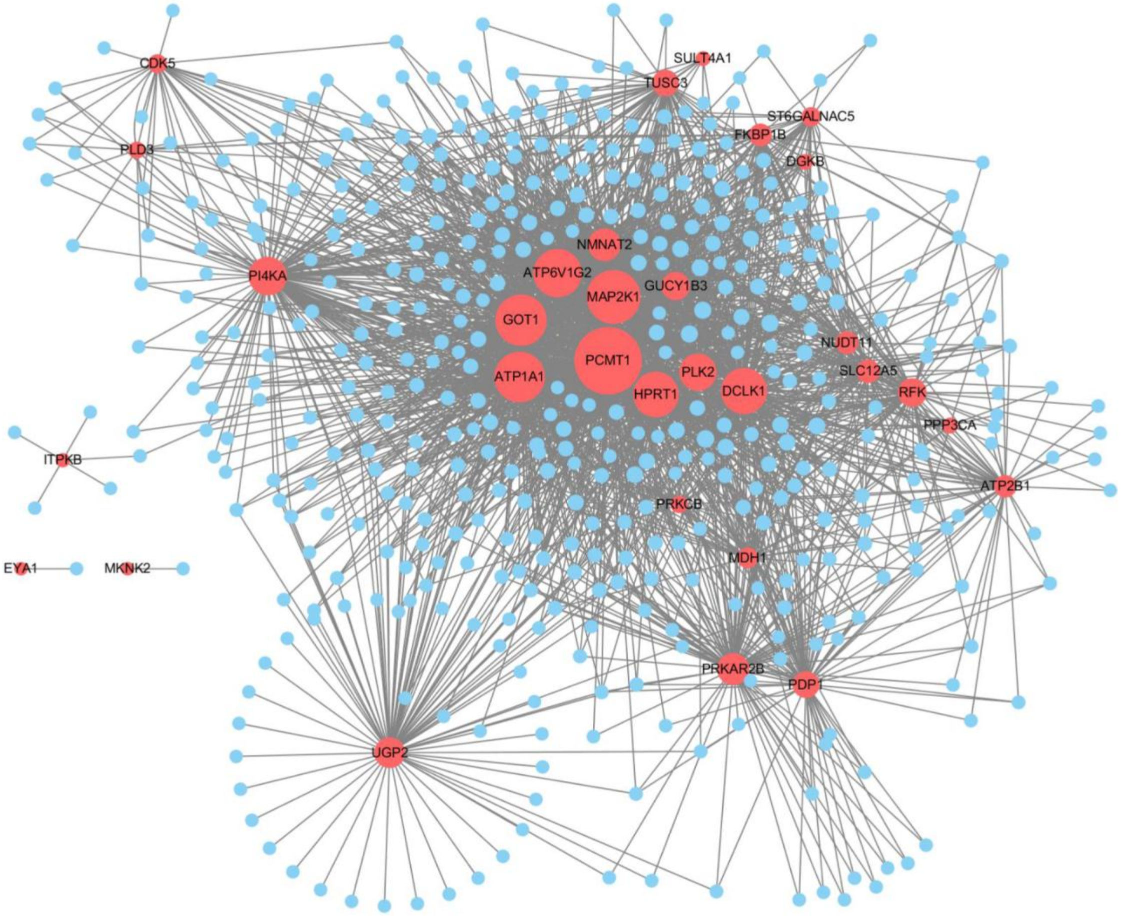

Supplementary Figure 12. Co-expression network of metabolic genes in Alzheimer's disease. The red circle represents deregulated gene shared by three neurodegenerative diseases and the cyan circle represents other metabolic genes.

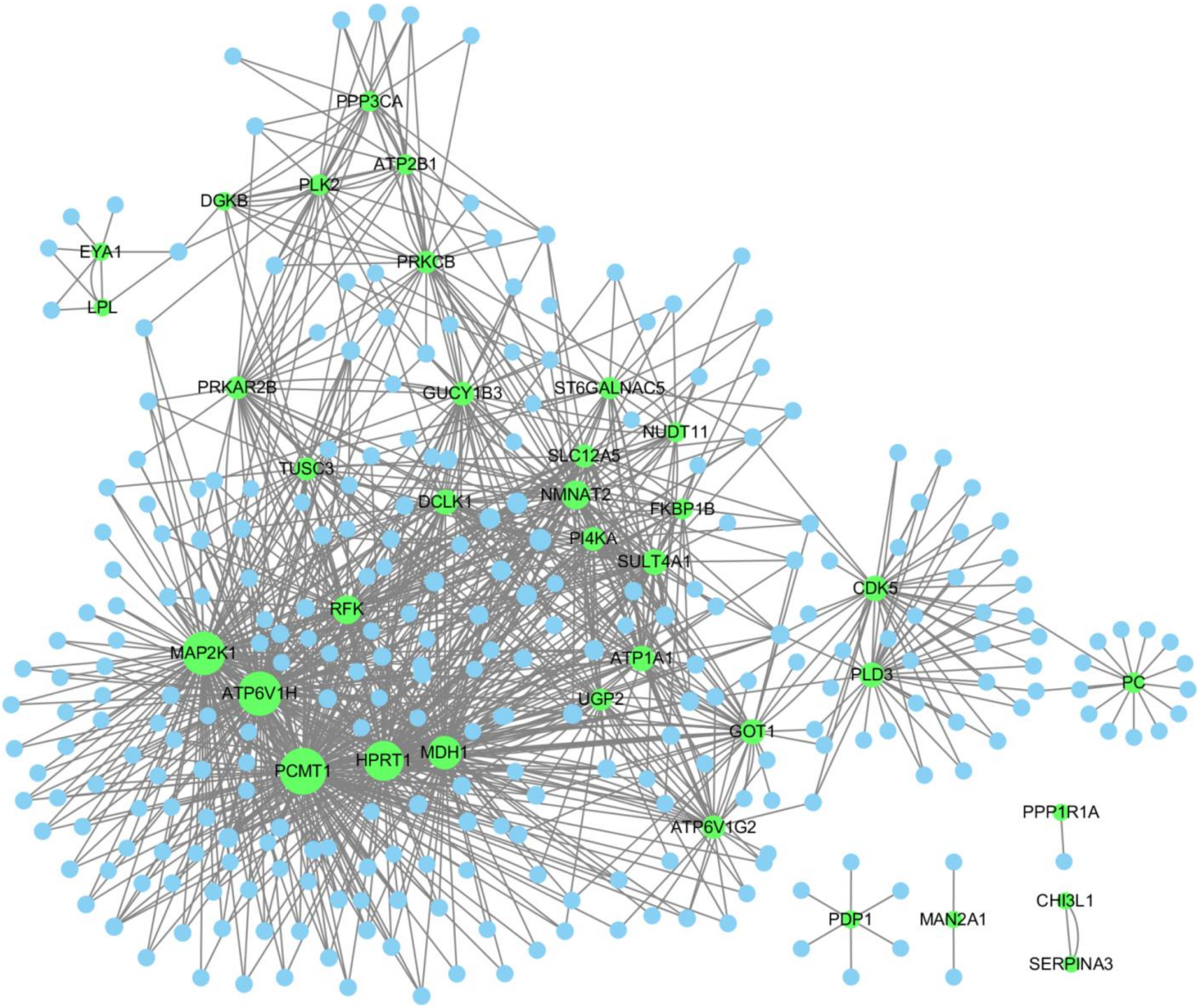

Supplementary Figure 13. Co-expression network of metabolic genes in Parkinson's disease. The green circle represents deregulated gene shared by three neurodegenerative diseases and the cyan circle represents other metabolic genes.

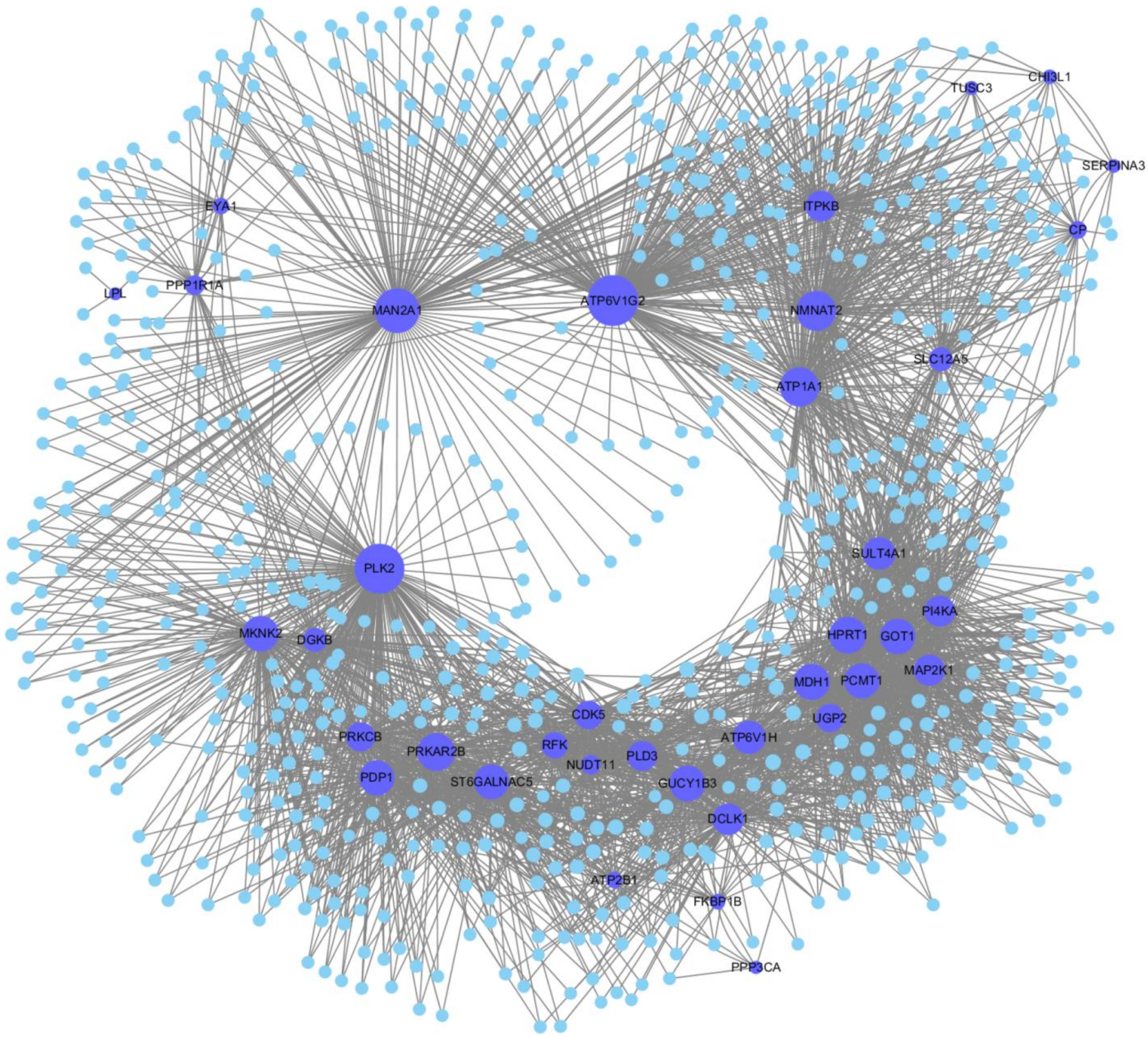

Supplementary Figure 14. Co-expression network of metabolic genes in Huntington's disease. The blue circle represents deregulated gene shared by three neurodegenerative diseases and the cyan circle represents other metabolic genes.

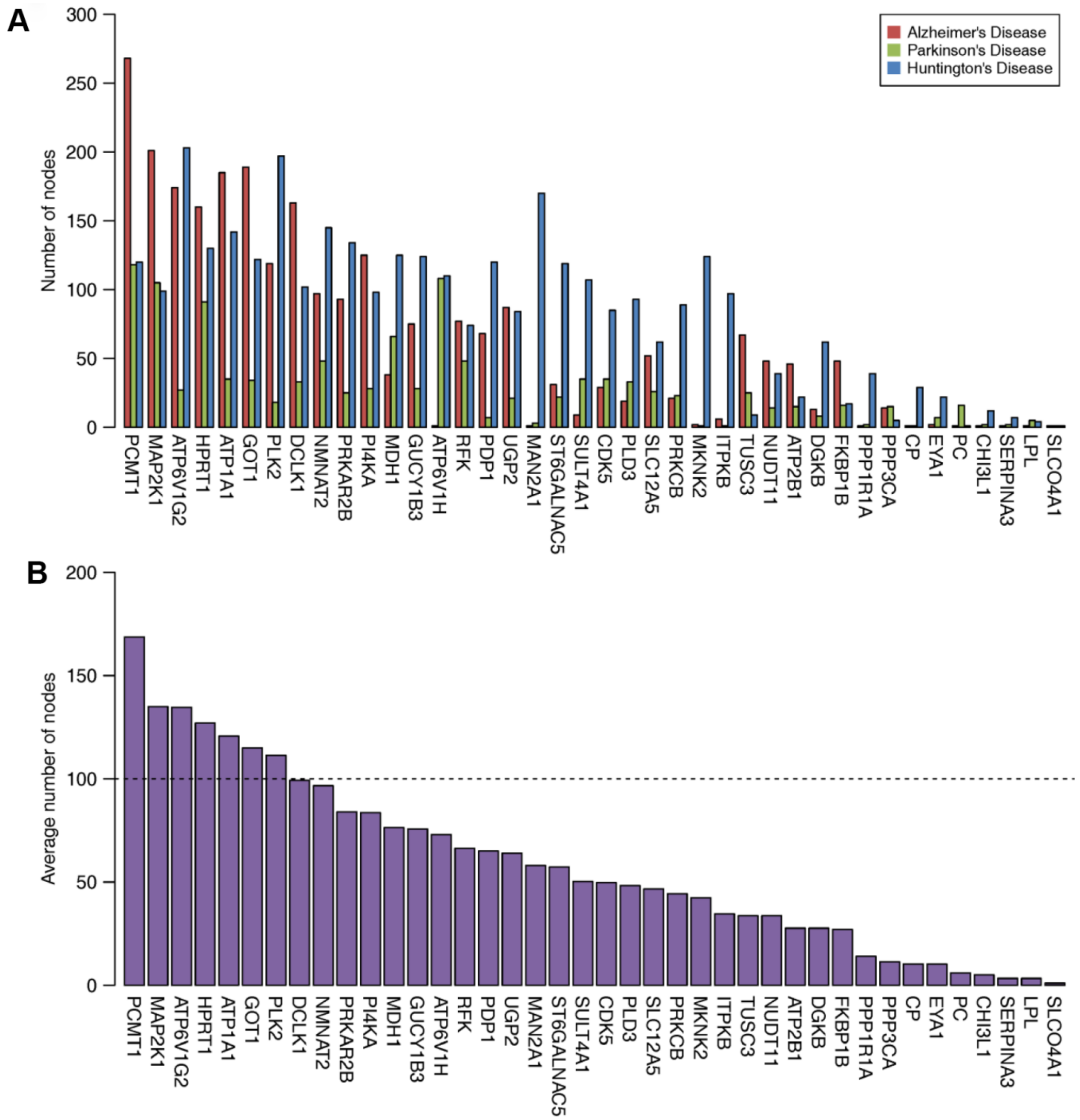

Supplementary Figure 15. Number of nodes of deregulated genes shared by three neurodegenerative diseases in gene co-expression networks. (**A**) Number of nodes of metabolic genes in each disease. (**B**) Average number of nodes of metabolic genes in AD, PD and HD.

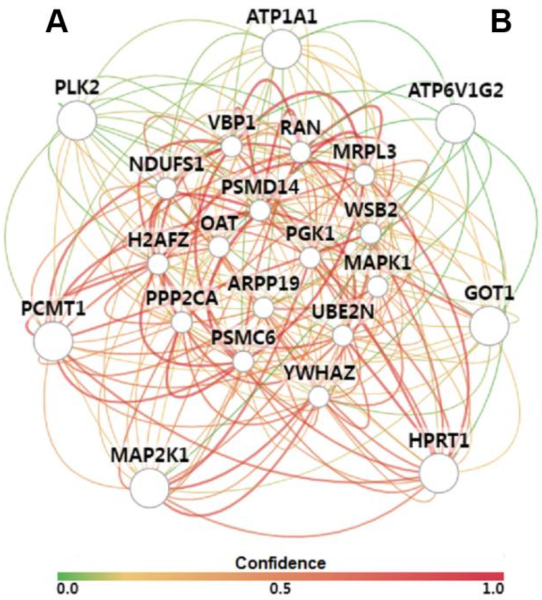

B

GOBP	FDR
purine ribonucleotide metabolic process	2.00E-02
ribonucleotide metabolic process	2.00E-02
purine nucleotide metabolic process	2.00E-02
ribose phosphate metabolic process	2.00E-02
purine-containing compound metabolic process	2.00E-02
purine ribonucleoside triphosphate metabolic process	2.00E-02
nucleotide metabolic process	2.00E-02
purine nucleoside monophosphate metabolic process	2.00E-02
purine ribonucleoside monophosphate metabolic process	2.00E-02
ribonucleoside triphosphate metabolic process	2.00E-02
nucleoside phosphate metabolic process	2.00E-02
ribonucleoside monophosphate metabolic process	2.00E-02
guanosine-containing compound metabolic process	2.00E-02
purine nucleoside triphosphate metabolic process	2.00E-02
nucleoside monophosphate metabolic process	2.50E-02
nucleoside triphosphate metabolic process	2.50E-02
purine ribonucleoside metabolic process	2.50E-02
nucleobase-containing small molecule metabolic process	2.50E-02
cellular response to organic cyclic compound	2.50E-02
purine nucleoside metabolic process	3.00E-02
ribonucleoside metabolic process	4.70E-02

Supplementary Figure 16. Brain specific network of key metabolic genes. (**A**) Network of key metabolic genes and correlated genes. (**B**) Enriched GO biological process of genes in the network.

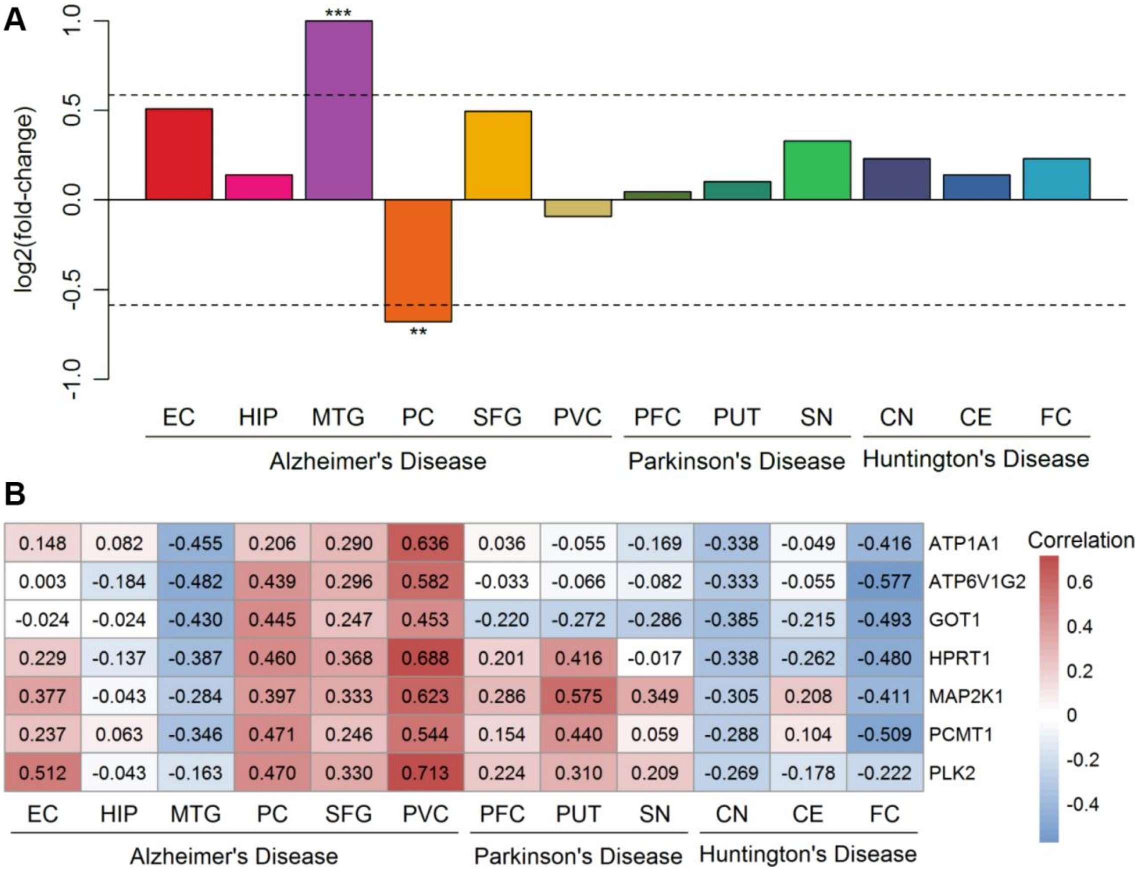

Supplementary Figure 17. Expression of SIRT1 in different brain regions (**A**) and its correlation with key metabolic genes (**B**). The color bar indicates the Pearson's correlation coefficient between SIRT1 and key metabolic genes. Statistical significance: * P < 0.05, ** P < 0.01, *** P < 0.001.

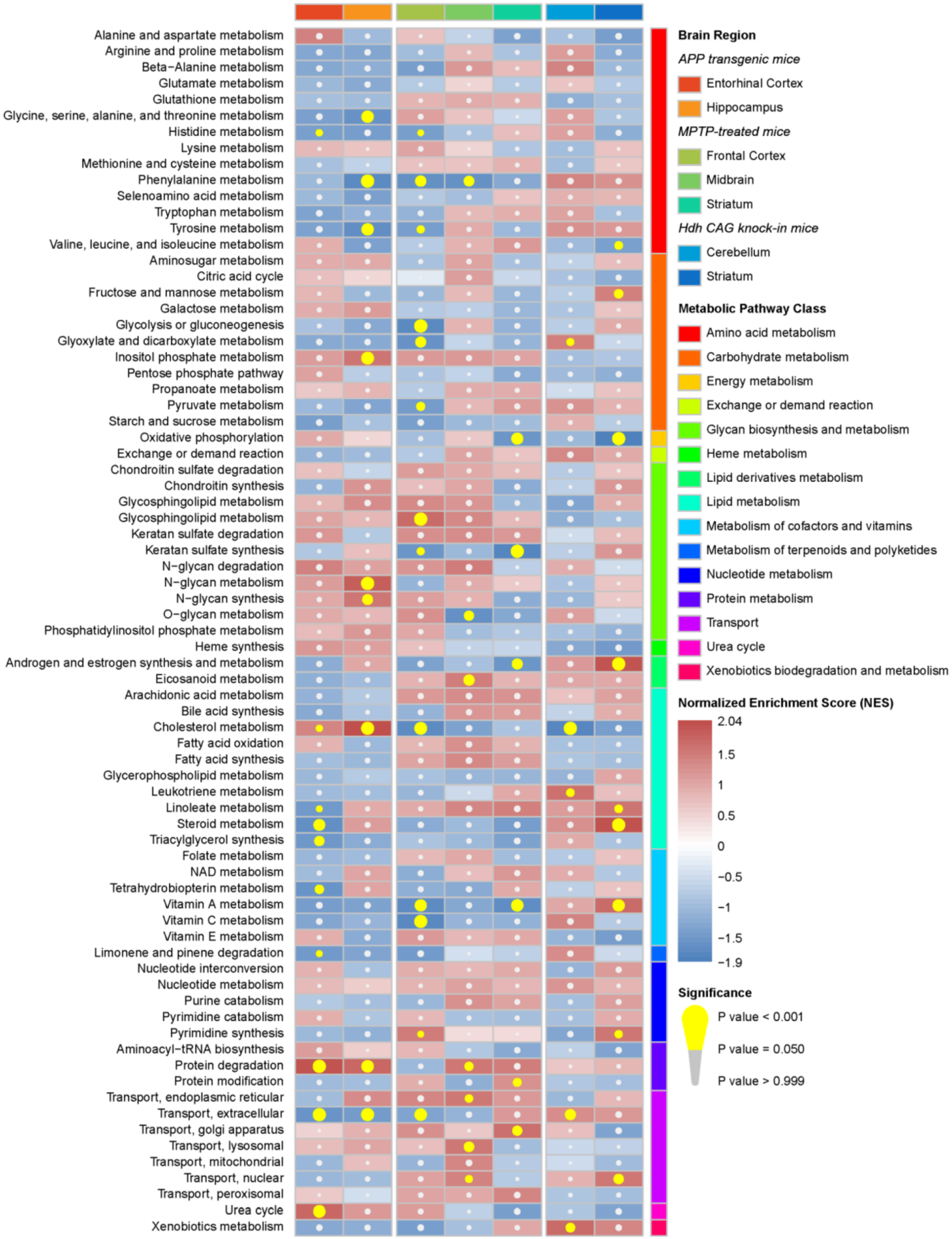

Supplementary Figure 18. Metabolic pathway enrichment results in three neurodegenerative diseases in mouse models. The red box represents the metabolic pathway is up-regulated and the blue box represents the metabolic pathway is down-regulated. The yellow circle indicates the metabolic pathway is significantly enriched.

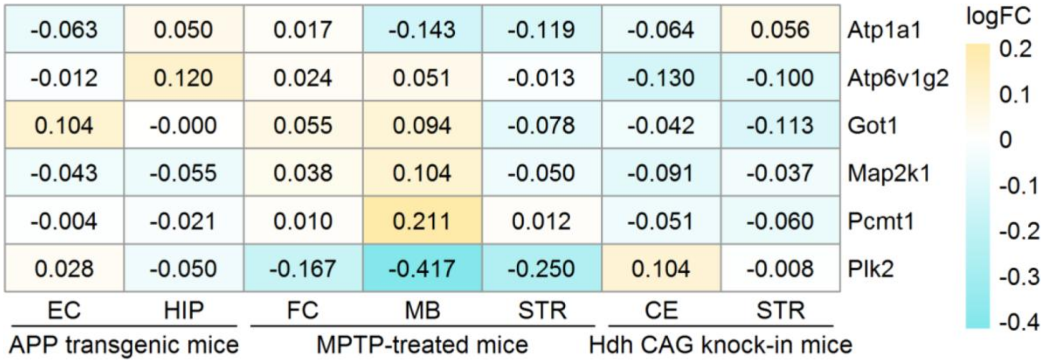

Supplementary Figure 19. Expression of key metabolic genes in mouse models. The heatmap showed the logFC of key metabolic genes in each brain region in mouse models. No Hrrt1 gene in mouse models datasets.
