# Supplementary material for: Systematic metabolic analysis of potential target, therapeutic drug, diagnostic method and animal model applicability in three neurodegenerative diseases
Source: Aging (Albany NY). 2020 May 27;12(10):9882–914. doi: 10.18632/aging.103253 (PMC7288927; doi:10.18632/aging.103253)
Supplement: Supplementary Tables 1, 2, 4 [file aging-12-103253-s002..pdf]

SUPPLEMENTARY TABLES

Supplementary Table 1. Information on the human brain transcriptome datasets of AD, PD and HD.[1]

GEO ID	Samples	Brain region	Platform	Mean PMI
GSE5281 (AD)	87 cases<br>74 controls	Entorhinal cortex<br>Hippocampus<br>Medial temporal gyrus<br>Posterior cingulate<br>Superior frontal gyrus<br>Primary visual cortex	Affymetrix Human Genome U133 Plus 2.0 Array	All: 2.5 h
GSE20295 (PD)	40 cases<br>53 controls	Prefrontal cortex<br>Putamen<br>Substantia nigra	Affymetrix Human Genome U133A Array	Case: 14.0 h<br>Control: 17.1 h
GSE3790 (HD)	114 cases<br>87 controls	Caudate nucleus<br>Cerebellum<br>Frontal cortex	Affymetrix Human Genome U133A Array	No information

[1] All datasets were conducted whole genome microarray expression test using postmortem brain tissues.
Abbreviations: AD: Alzheimer's disease, PD: Parkinson's disease, HD: Huntington's disease, PMI: postmortem interval.

Supplementary Table 2. Information on the human blood transcriptome datasets of AD, PD and HD.

GEO ID	Samples	Tissue	Platform
Experimental dataset			
GSE63060 (AD)	145 cases<br>104 controls	Blood	Illumina HumanHT-12 V3.0 expression beadchip
GSE99039 (PD)	205 cases<br>233 controls	Blood	Affymetrix Human Genome U133 Plus 2.0 Array
GSE51799 (HD)	91 cases<br>33 controls	Blood	Illumina Genome Analyzer IIx (Homo sapiens)<br>Illumina HiSeq 2000 (Homo sapiens)
Validation dataset			
GSE63061 (AD)	140 cases<br>134 controls	Blood	Illumina HumanHT-12 V4.0 expression beadchip
GSE57475 (PD)	93 cases<br>49 controls	Blood	Illumina HumanHT-12 V3.0 expression beadchip
GSE1751 (HD)	12 cases<br>14 controls	Blood	Affymetrix Human Genome U133A Array

Abbreviations: AD: Alzheimer's disease, PD: Parkinson's disease, HD: Huntington's disease.

Supplementary Table 4. Information on the mouse brain transcriptome datasets of AD, PD and HD.

GEO ID	Samples	Brain region	Platform	Animal model
GSE14499 (AD)	16 cases<br>10 controls	Entorhinal cortex<br>Hippocampus	Affymetrix Mouse Genome 430 2.0 Array	APP transgenic mice
GSE7707 (PD)	9 cases<br>9 controls	Frontal cortex<br>Midbrain<br>Striatum	Affymetrix Mouse Genome 430 2.0 Array	MPTP-treated mice
GSE9038 (HD)	12 cases<br>12 controls	Cerebellum<br>Striatum	Affymetrix Mouse Genome 430 2.0 Array	Hdh CAG knock-in mice

Abbreviations: AD: Alzheimer's disease, PD: Parkinson's disease, HD: Huntington's disease.
