# Supplementary material for: Systematic metabolic analysis of potential target, therapeutic drug, diagnostic method and animal model applicability in three neurodegenerative diseases
Source: Aging (Albany NY). 2020 May 27;12(10):9882–914. doi: 10.18632/aging.103253 (PMC7288927; doi:10.18632/aging.103253)
Supplement: Supplementary Table 3 [file aging-12-103253-s001..docx]

**Supplementary Table 3.** Name and indication information of the screened 57 drugs.

| **DrugBank ID** | **Name** | **Indication/Associated Conditions** |
| --- | --- | --- |
| DB00390 | Digoxin | Chronic atrial fibrillation Mild to moderate heart failure |
| DB00511 | Acetyldigitoxin | Congestive heart failure |
| DB01078 | Deslanoside | Congestive cardiac insufficiency Arrhythmias Heart failure |
| DB01092 | Ouabain | Atrial fibrillation Heart failure |
| DB00774 | Hydroflumethiazide | Congestive heart failure Hepatic cirrhosis Hypertension |
| DB01021 | Trichlormethiazide | Oedema Hypertension |
| DB01396 | Digitoxin | Congestive cardiac insufficiency Arrhythmias Heart failure |
| DB00903 | Etacrynic acid | Ascites Congenital heart disease Edema Nephrotic syndrome |
| DB01119 | Diazoxide | Hyperinsulinemic hypoglycemia |
| DB01158 | Bretylium | Ventricular fibrillation Ventricular tachycardia |
| DB01244 | Bepridil | Hypertension Chronic stable angina |
| DB01345 | Potassium cation | Hypokalemia |
| DB01370 | Aluminium | Not Available |
| DB01378 | Magnesium cation | Not Available |
| DB01430 | Almitrine | Chronic obstructive pulmonary disease |
| DB06157 | Istaroxime | Heart disease |
| DB01188 | Ciclopirox | Candidiasis, cutaneous Pityriasis versicolor Seborrheic dermatitis of the scalp Tinea corporis Tinea cruris Tinea pedis Mild onychomycosis Moderate onychomycosis |
| DB09479 | Rubidium Rb-82 | Coronary artery disease |
| DB13996 | Magnesium acetate | Not Available |
| DB13749 | Magnesium gluconate | Low levels of magnesium |
| DB13620 | Potassium gluconate | Hypokalemia |
| DB14498 | Potassium acetate | Hypokalemia |
| DB14499 | Potassium sulfate | Hypokalemia |
| DB14514 | Magnesium levulinate | Not Available |
| DB14515 | Magnesium lactate | Not Available |
| DB14517 | Aluminium phosphate | Not Available |
| DB14518 | Aluminum acetate | Outer ear infection |
| DB01133 | Tiludronic acid | Paget's disease of bone |
| DB00630 | Alendronic acid | Osteogenesis imperfecta Osteoporosis Osteoporosis caused by glucocorticoid Paget's disease |
| DB01077 | Etidronic acid | Paget's disease of bone Heterotropic ossification |
| DB06733 | Bafilomycin A1 | Not Available |
| DB06734 | Bafilomycin B1 | Not Available |
| DB00114 | Pyridoxal phosphate | Pernicious anemia |
| DB00128 | Aspartic acid | Ergogenic aids |
| DB00151 | Cysteine | Liver damage and kidney damage |
| DB00142 | Glutamic acid | Nutritional supplement |
| DB04299 | Maleic Acid | Not Available |
| DB09130 | Copper | Supplementation of total parenteral nutrition Contraception |
| DB01632 | 5-O-phosphono-alpha-D-ribofuranosyl diphosphate | Not Available |
| DB02309 | 5-monophosphate-9-beta-D-ribofuranosyl xanthine | Not Available |
| DB04356 | 9-Deazaguanine | Not Available |
| DB03153 | 3H-pyrazolo[4,3-d]pyrimidin-7-ol | Not Available |
| DB00993 | Azathioprine | Atopic dermatitis Crohn's disease Disseminated sclerosis Immune thrombocytopenia Kidney transplant rejection Nephritis, lupus Pericarditis Psoriasis Rheumatoid arthritis Uveitis |
| DB01033 | Mercaptopurine | Acute lymphoblastic leukaemias Acute promyelocytic leukemia Crohn's disease Hepatitis, autoimmune Lymphoma, lymphoblastic Ulcerative colitis |
| DB00352 | Tioguanine | Acute nonlymphocytic leukemia |
| DB03115 | 5-Bromo-N-[(2S)-2,3-dihydroxypropoxy]-3,4-difluoro-2-[(2-fluoro-4-iodophenyl)amino]benzamide | Not Available |
| DB05239 | Cobimetinib | Melanoma |
| DB06892 | (5S)-4,5-difluoro-6-[(2-fluoro-4-iodophenyl)imino]-N-(2-hydroxyethoxy)cyclohexa-1,3-diene-1-carboxamide | Not Available |
| DB07046 | 2-[(2-chloro-4-iodophenyl)amino]-N-{[(2R)-2,3-dihydroxypropyl]oxy}-3,4-difluorobenzamide | Not Available |
| DB07101 | PD-0325901 | Not Available |
| DB02152 | K-252a | Not Available |
| DB08130 | N-(5-{3,4-difluoro-2-[(2-fluoro-4-iodophenyl)amino]phenyl}-1,3,4-oxadiazol-2-yl)ethane-1,2-diamine | Not Available |
| DB08208 | 2-[(4-ethynyl-2-fluorophenyl)amino]-3,4-difluoro-n-(2-hydroxyethoxy)benzamide | Not Available |
| DB06616 | Bosutinib | Chronic myelogenous leukemia |
| DB08911 | Trametinib | Melanoma |
| DB01752 | S-adenosyl-L-homocysteine | Not Available |
| DB12010 | Fostamatinib | Chronic immune thrombocytopenia |
